# Supplementary figures and images for: AMPK-dependent and -independent coordination of mitochondrial function and muscle fiber type by FNIP1
Source: PLoS Genet. 2021 Mar 29;17(3):e1009488. doi: 10.1371/journal.pgen.1009488 (PMC8031738; doi:10.1371/journal.pgen.1009488)

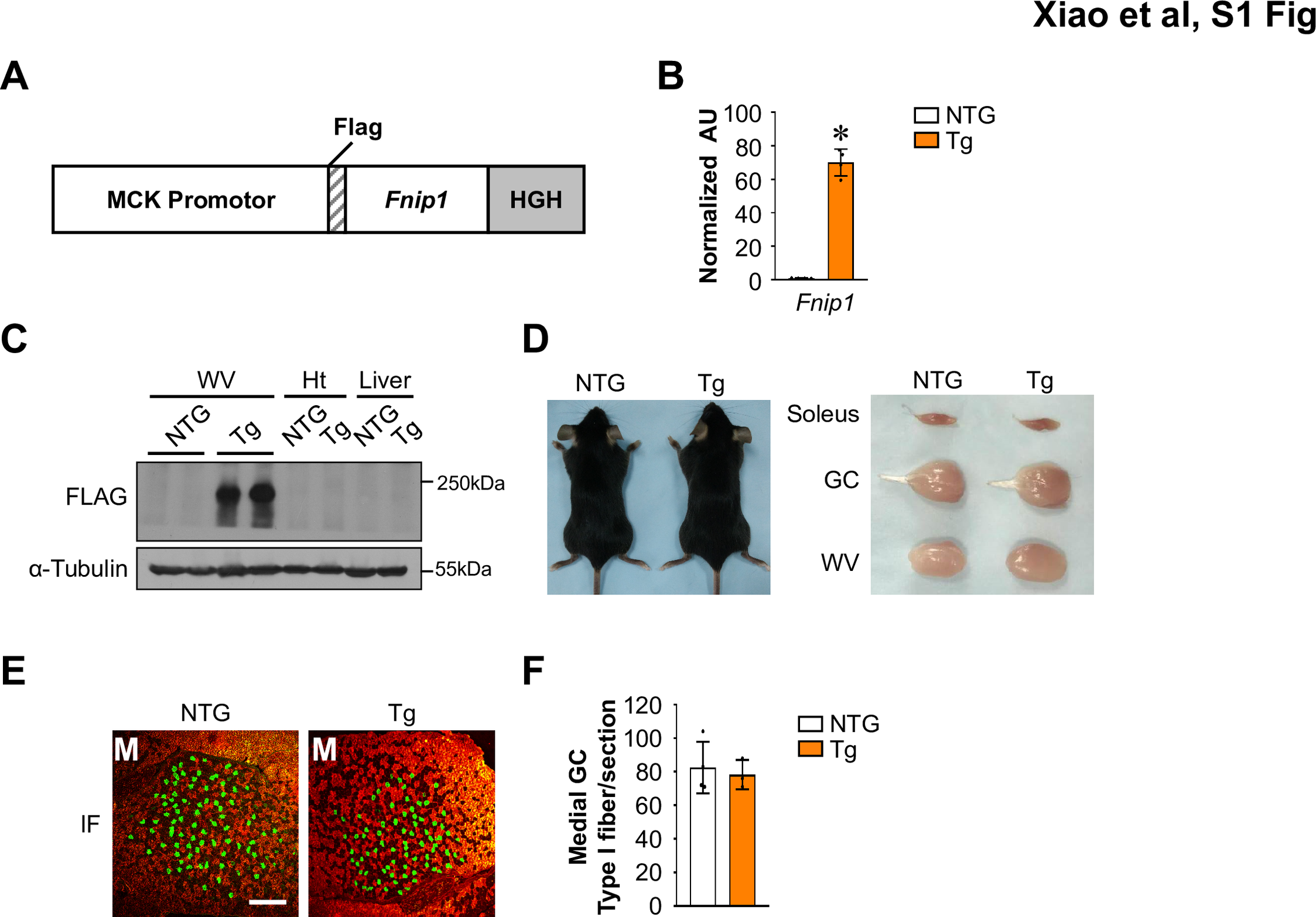

Supplement: S1 Fig — (A) The schematic depicts the Mck-Fnip1 construct used for Fnip1Tg transgene production. (B) RT-qPCR analysis of Fnip1 mRNA levels in the entire gastrocnemius (GC) muscle from nontransgenic (NTG) and Fnip1Tg mice. n = 4 mice per group. (C) Representative immunoblot analysis of protein extracts prepared from white vastus lateralis (WV) muscles, heart (Ht) and liver of the indicated mice using FLAG and ɑ-Tubulin (control) antibodies. n = 4 mice per group. (D) (Left) Pictures of NTG and Fnip1Tg mice at the age of 8 weeks. (Right) Representative soleus, GC and WV muscles from indicated mice. (E) Cross-section of GC muscle from 8-week-old male NTG and Fnip1Tg mice stained for MHC immunofluorescence (IF). Representative images were shown. M (medial head of GC), MHC1 (green), and MHC2b (red). Scale bar: 300 μm. (F) Quantification of IF data shown in (E). n = 3–4 mice per group. Values represent mean ± SD, *P < 0.05 vs. NTG controls. (TIF) [file pgen.1009488.s001.tif]

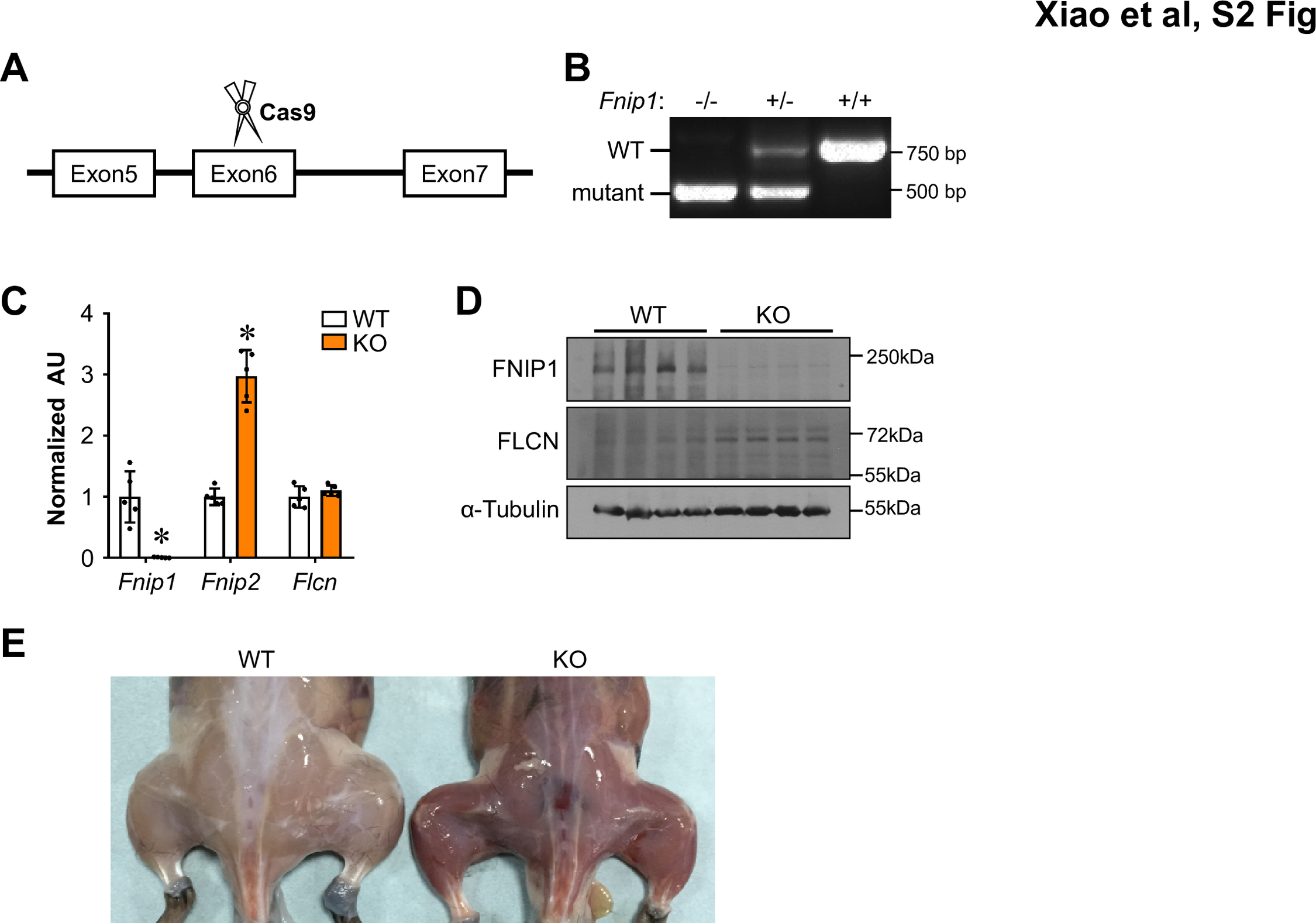

Supplement: S2 Fig — (A) Schematic showing the Cas9 system targeting exon 6 of the Fnip1 gene to generate Fnip1 knockout mice (Fnip1KO). (B) Detection of the Fnip1 mutation by PCR. Primers flanking the exon 6 of the Fnip1 gene generate PCR products as indicated. (C) Results of RT-qPCR analysis of Fnip1, Fnip2 and Flcn mRNA levels in entire GC muscles from indicated mice. n = 5 mice per group. (D) Immunoblot analysis of protein extracts prepared from WV muscles of the indicated mice using FNIP1, FLCN and ɑ-Tubulin (control) antibodies. n = 4 mice per group. (E) Representative pictures showing WT and Fnip1KO mice skeletal muscle at the age of 8 weeks. Values represent mean ± SD, *P < 0.05 vs. WT controls. (TIF) [file pgen.1009488.s002.tif]

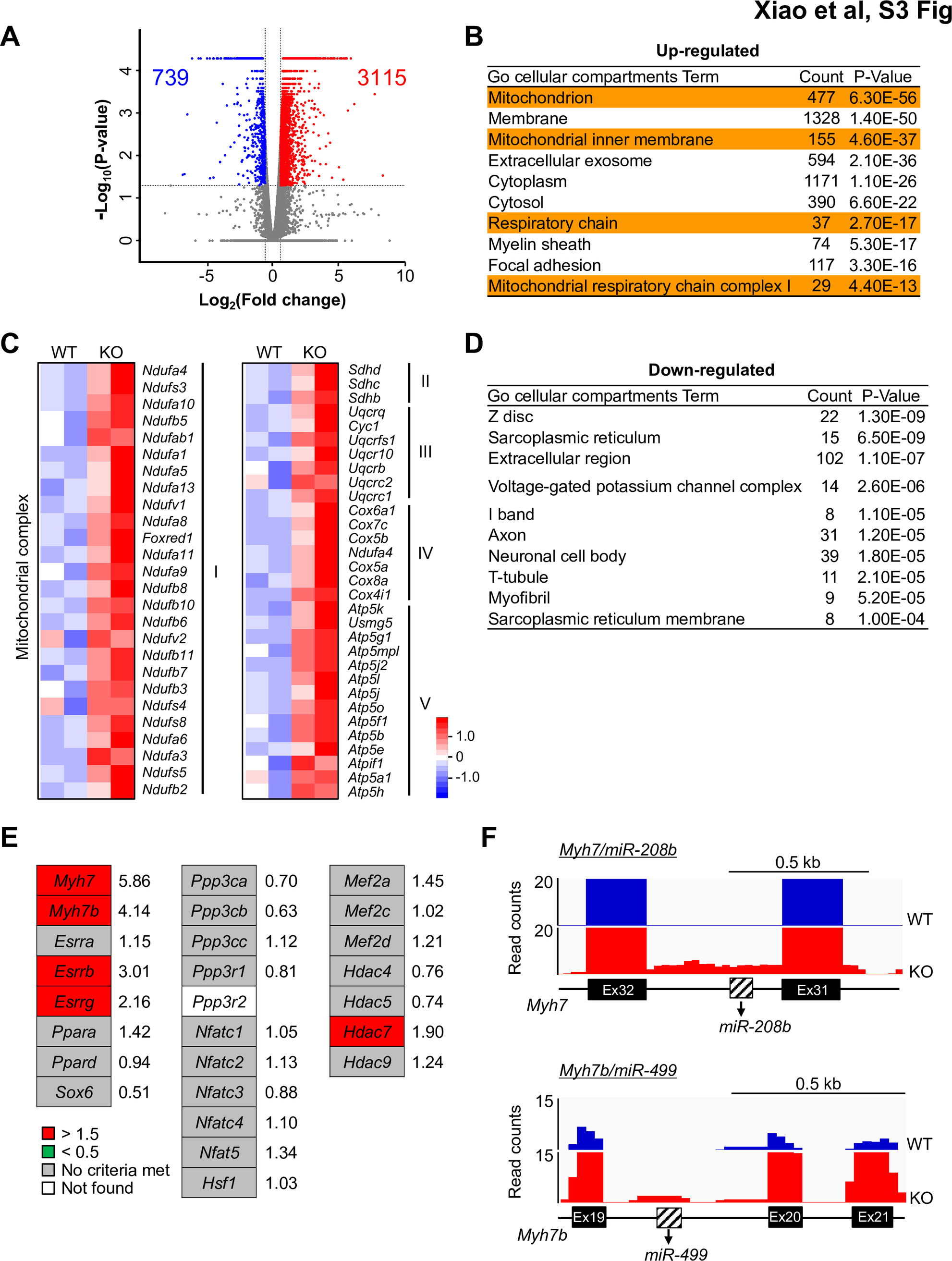

Supplement: S3 Fig — (A) Volcano plot showing fold changes versus P-values for the analyzed RNA-seq data generated from the entire GC muscle of 8-week-old male Fnip1KO mice compared to WT controls. Significantly up-regulated genes are represented by red dots, whereas down-regulated genes are represented by blue dots. (B) Gene ontology (GO) enrichment analysis of gene transcripts (top 3000) up-regulated in Fnip1KO muscle, with the top ten terms shown. (C) Heat-map of up-regulated mitochondrial complex genes in Fnip1KO muscles, color scheme for fold change is provided. (D) GO enrichment analysis of 739 gene transcripts down-regulated in Fnip1KO muscle. (E) Heat maps depicting a subset of gene expression data, individual genes involved in the regulation of type I muscle fiber specification are shown to be regulated in the Fnip1KO as denoted by the color scheme (relative fold change compared to WT control shown to the right of each gene). (F) RNA-seq data of WT and Fnip1KO muscle indicate an increase expression of miR-208b and miR-499. (TIF) [file pgen.1009488.s003.tif]

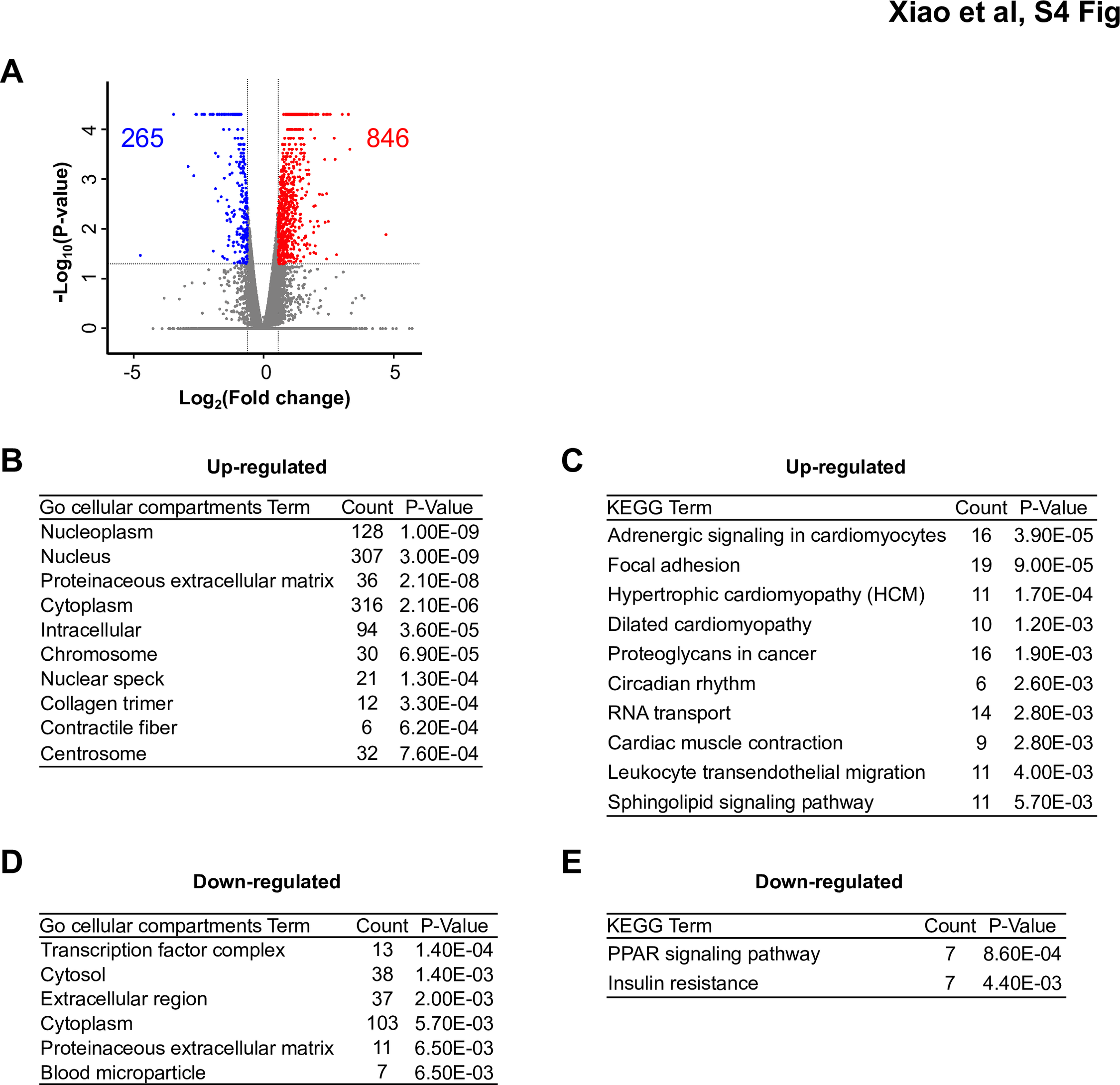

Supplement: S4 Fig — (A) Volcano plot showing fold changes versus P-values for the analyzed RNA-seq data generated from the entire GC muscle of 8-week-old male Fnip1TgKO mice compared to WT controls. Significantly up-regulated genes are represented by red dots, whereas down-regulated genes are represented by blue dots. (B, C) GO enrichment analysis of gene transcripts up-regulated in Fnip1TgKO muscle. (D, E) GO enrichment analysis of gene transcripts down-regulated in Fnip1TgKO muscle. (TIF) [file pgen.1009488.s004.tif]

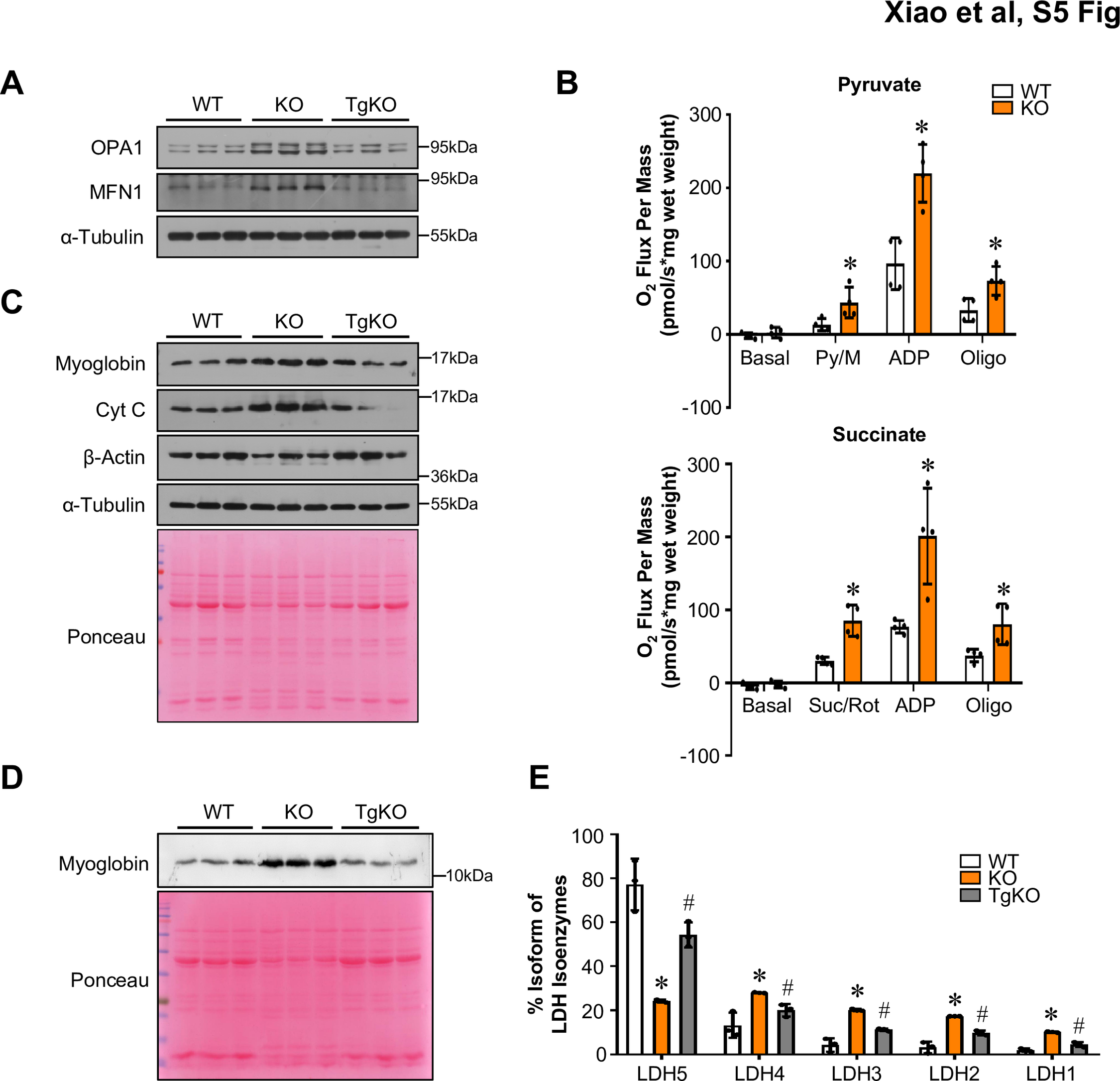

Supplement: S5 Fig — (A, C) Immunoblot analysis of entire GC muscle lysates for mice of the indicated genotype using the indicated antibodies. Ponceau red staining was also shown. n = 3 mice per group. (B) Mitochondrial respiration rates were determined from the plantaris part of the gastrocnemius/plantaris complex of the indicated genotypes using pyruvate or succinate as substrates. Pyruvate/malate (Py/M) or succinate/rotenone (Suc/Rot)-stimulated, ADP-dependent respiration and oligomycin-induced (Oligo) are shown. n = 4 mice per group. (D) (Top) Immunoblot analysis of WV muscle lysates for mice of the indicated genotype using the myoglobin antibody. (Bottom) Ponceau red staining was shown. n = 3 mice per group. (E) Quantification of LDH isoenzyme activity gel electrophoresis. Values represent the mean % (+/- SD) total LDH activity. n = 3 mice per group. Values represent mean ± SD, *P < 0.05 vs. WT controls, # P < 0.05 vs. Fnip1KO. (TIF) [file pgen.1009488.s005.tif]

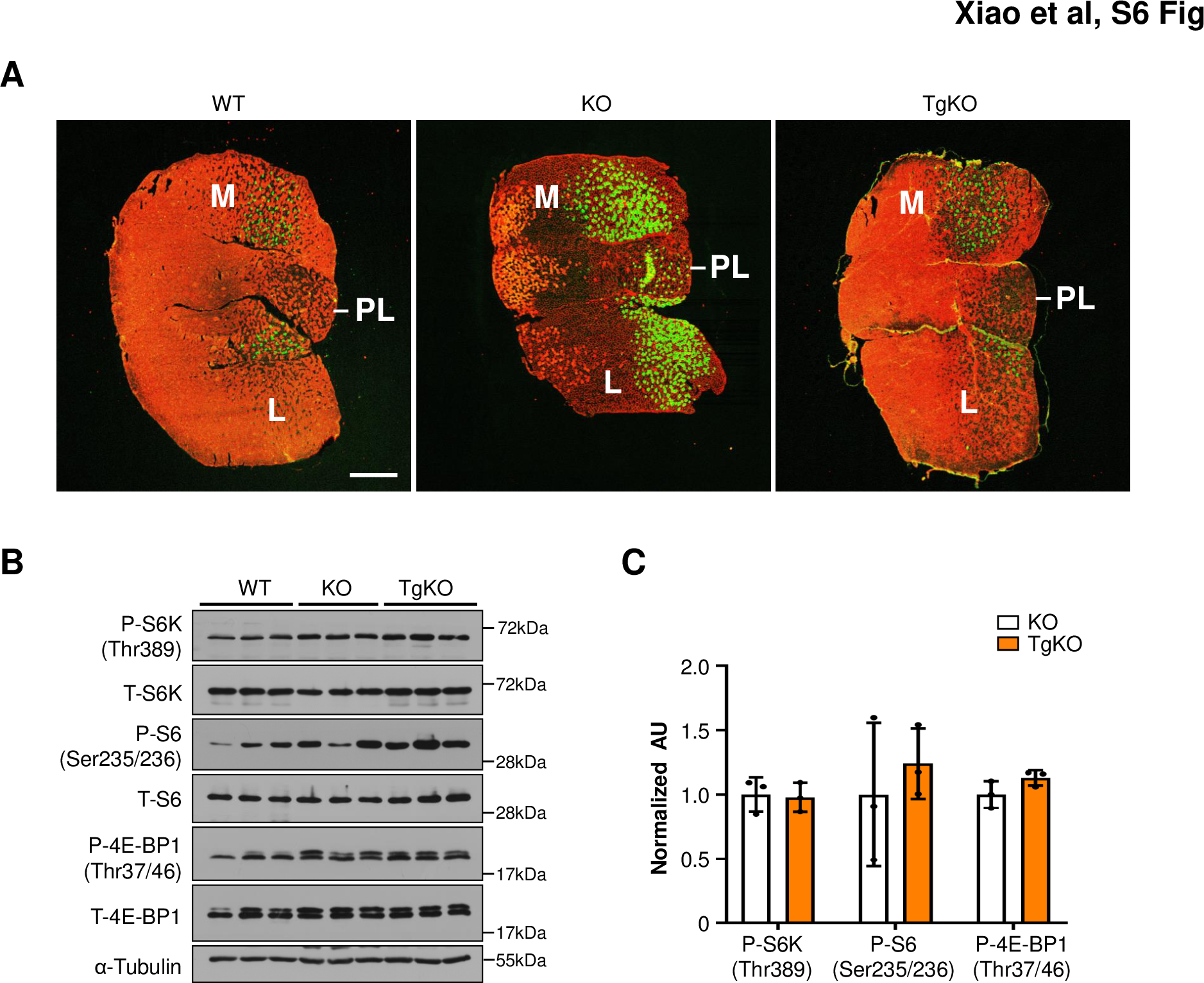

Supplement: S6 Fig — (A) Low power MHC immunofluorescence scan showing entire cross-section of the mid-belly GC muscle of WT, Fnip1KO and Fnip1TgKO mice at the age of 8 weeks. (M) medial; (L) lateral; (PL) plantaris. MHC1 (green), and MHC2b (red). Scale bar: 1000 μm. (B) Immunoblot analysis of WV muscle lysates for mice of the indicated genotype using the indicated antibodies. (C) Quantification of the p-S6K (Thr389)/S6K, p-S6 (Ser235/236)/S6 and p-4EBP1 (Thr37/46)/4EBP1 signal ratios were normalized (= 1.0) to the Fnip1KO. n = 3 mice per group. (TIF) [file pgen.1009488.s006.tif]

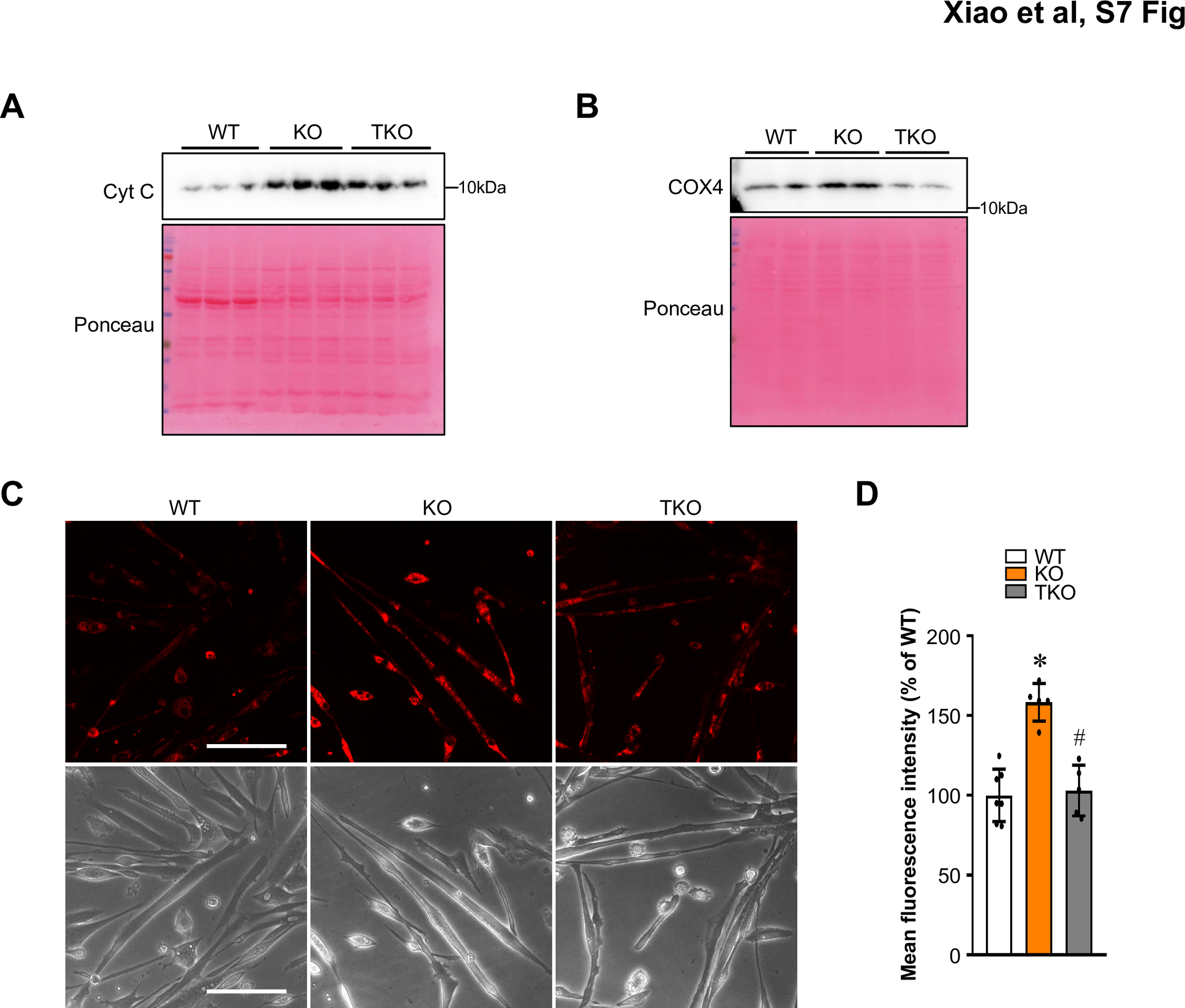

Supplement: S7 Fig — (A) (Top) Immunoblot analysis of muscle lysates for mice of the indicated genotype using the cytochrome c antibody. (Bottom) Ponceau red staining was shown. n = 3 mice per group. (B-D) Primary skeletal muscle myoblasts were isolated from GC muscles of WT, Fnip1KO, or AMPKa1/a2f/f/Myf5-Cre (TKO) mice, and then induced to differentiation for 3 days. (B) (Top) Immunoblot analysis of myotube extracts using the COX4 antibody. (Bottom) Ponceau red staining was shown. (C) (Top) Myotubes were stained with MitoTracker Red CMXRos, (Bottom) Phase contrast microscopy images of myotubes were shown. Scale bars: 100 μm. (D) Quantification of mitotracker fluorescent signal intensity in (C). n = 3 independent experiments. Values represent mean ± SD, *P < 0.05 vs. WT controls, # P < 0.05 vs. Fnip1KO. (TIF) [file pgen.1009488.s007.tif]

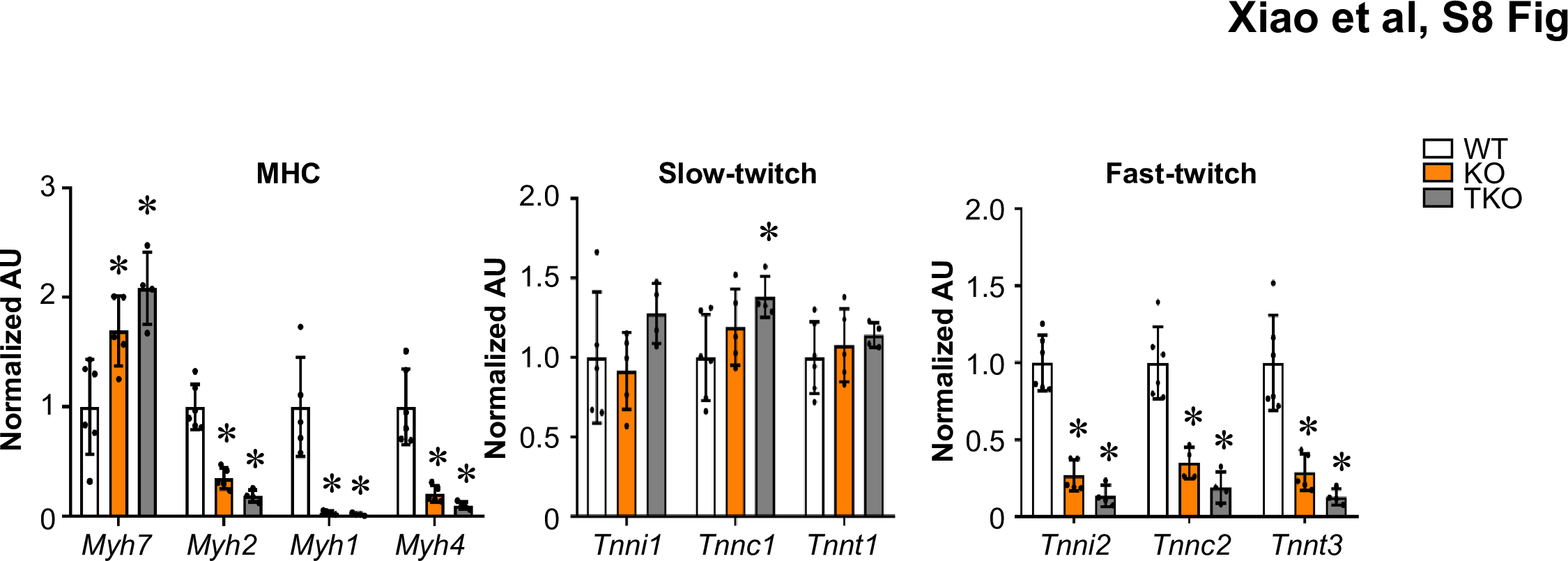

Supplement: S8 Fig — Expression of myosin heavy chain (MHC) and representative slow/fast-twitch troponin genes (qRT-PCR) in soleus muscles from indicated mice. n = 4–6 mice per group. Values represent mean ± SD, *P < 0.05 vs. WT controls. (TIF) [file pgen.1009488.s008.tif]

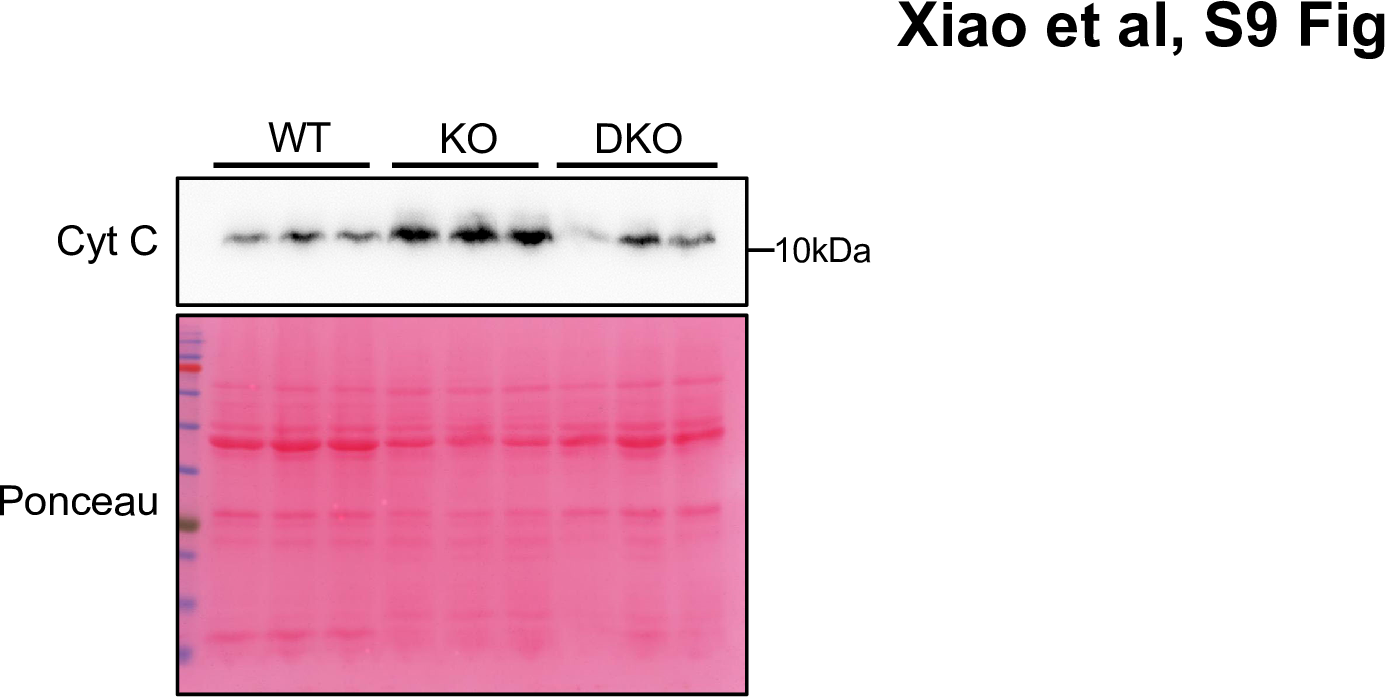

Supplement: S9 Fig — (Top) Immunoblot analysis of entire GC muscle lysates for mice of the indicated genotype using the cytochrome c antibody. (Bottom) Ponceau red staining was shown. n = 3 mice per group. (TIF) [file pgen.1009488.s009.tif]
