# Supplementary material for: AMPK-dependent and -independent coordination of mitochondrial function and muscle fiber type by FNIP1
Source: PLoS Genet. 2021 Mar 29;17(3):e1009488. doi: 10.1371/journal.pgen.1009488 (PMC8031738; doi:10.1371/journal.pgen.1009488)
Supplement: S1 Table — (DOCX) [file pgen.1009488.s010.docx]

**S1 Table. Body weight and muscle weight measurements**

|  | *WT* | *KO* | *TgKO* |
| --- | --- | --- | --- |
| *Body weight (g)* | *25.6 ± 2.0* | *20.6 ± 2.8** | *24.5 ± 0.8* |
| *Gastrocnemius weight (g)* | *0.143 ± 0.0063* | *0.096 ± 0.0086** | *0.141 ± 0.0091* |
| *Relative gastrocnemius weight (%)* | *0.559 ± 0.0278* | *0.471 ± 0.0697** | *0.574 ± 0.0454* |

Body weight and muscle weight of male WT, *Fnip1*^KO^ and *Fnip1*^TgKO^ mice were measured at the age of 12 weeks. Data represent the mean ± SD; n = 4-6 mice per group; **P* < 0.05 versus WT.
