# Supplementary material for: AMPK-dependent and -independent coordination of mitochondrial function and muscle fiber type by FNIP1
Source: PLoS Genet. 2021 Mar 29;17(3):e1009488. doi: 10.1371/journal.pgen.1009488 (PMC8031738; doi:10.1371/journal.pgen.1009488)
Supplement: S2 Table — (DOCX) [file pgen.1009488.s011.docx]

**S2 Table. FNIP1-dependent mitochondrial-related genes (455)**

| ***Gene name*** | ***Description*** | ***Fold change***  ***(vs. WT)*** | ***P value*** |
| --- | --- | --- | --- |
| *Abcb10* | *ATP-binding cassette, sub-family B , member 10* | *1.74* | *0.00015* |
| *Abcd2* | *ATP-binding cassette, sub-family D, member 2* | *-3.60* | *5.00E-05* |
| *Abcg1* | *ATP-binding cassette, sub-family G, member 1* | *2.97* | *5.00E-05* |
| *Abcg2* | *ATP-binding cassette, sub-family G, member 2* | *3.60* | *5.00E-05* |
| *Abhd6* | *abhydrolase domain containing 6(Abhd6)* | *1.71* | *0.00835* |
| *Acaa2* | *acetyl-Coenzyme A acyltransferase 2* | *2.07* | *5.00E-05* |
| *Acaca* | *acetyl-Coenzyme A carboxylase alpha* | *5.57* | *5.00E-05* |
| *Acacb* | *acetyl-Coenzyme A carboxylase beta* | *1.86* | *0.0001* |
| *Acadl* | *acyl-Coenzyme A dehydrogenase, long-chain* | *2.31* | *5.00E-05* |
| *Acadsb* | *acyl-Coenzyme A dehydrogenase, short/branched chain* | *-2.30* | *5.00E-05* |
| *Acadvl* | *acyl-Coenzyme A dehydrogenase, very long chain* | *1.92* | *5.00E-05* |
| *Acly* | *ATP citrate lyase* | *2.44* | *5.00E-05* |
| *Aco1* | *aconitase 1* | *1.64* | *0.0012* |
| *Acot1* | *acyl-CoA thioesterase 1* | *7.51* | *5.00E-05* |
| *Acot13* | *acyl-CoA thioesterase 13* | *2.35* | *5.00E-05* |
| *Acot2* | *acyl-CoA thioesterase 2* | *2.78* | *5.00E-05* |
| *Acot9* | *acyl-CoA thioesterase 9* | *2.23* | *5.00E-05* |
| *Acox2* | *acyl-Coenzyme A oxidase 2, branched chain* | *10.90* | *0.0033* |
| *Acox3* | *acyl-Coenzyme A oxidase 3, pristanoyl* | *1.81* | *0.00025* |
| *Acsf2* | *acyl-CoA synthetase family member 2* | *6.82* | *0.0002* |
| *Acsf3* | *acyl-CoA synthetase family member 3* | *2.36* | *5.00E-05* |
| *Acsl1* | *acyl-CoA synthetase long-chain family member 1* | *2.37* | *5.00E-05* |
| *Acsl3* | *acyl-CoA synthetase long-chain family member 3* | *-3.20* | *0.00005* |
| *Acsl5* | *acyl-CoA synthetase long-chain family member 5* | *1.60* | *0.00855* |
| *Acsl6* | *acyl-CoA synthetase long-chain family member 6* | *3.51* | *5.00E-05* |
| *Acss1* | *acyl-CoA synthetase short-chain family member 1* | *2.50* | *5.00E-05* |
| *Acss3* | *acyl-CoA synthetase short-chain family member 3* | *-1.99* | *0.01835* |
| *Adam12* | *a disintegrin and metallopeptidase domain 12* | *2.50* | *0.0011* |
| *Adck1* | *aarF domain containing kinase 1* | *2.10* | *5.00E-05* |
| *Adh1* | *alcohol dehydrogenase 1* | *2.27* | *5.00E-05* |
| *Adh5* | *alcohol dehydrogenase 5, chi polypeptide* | *1.67* | *0.0003* |
| *Adhfe1* | *alcohol dehydrogenase, iron containing, 1* | *-3.06* | *5.00E-05* |
| *Agap2* | *ArfGAP with GTPase domain, ankyrin repeat and PH domain 2* | *2.00* | *0.0002* |
| *Aifm1* | *apoptosis-inducing factor, mitochondrion-associated 1* | *1.75* | *0.00015* |
| *Ak3* | *adenylate kinase 3* | *1.63* | *0.02015* |
| *Ak4* | *adenylate kinase 4* | *5.83* | *5.00E-05* |
| *Akap1* | *A kinase anchor protein 1* | *2.61* | *5.00E-05* |
| *Akt1* | *thymoma viral proto-oncogene 1* | *3.81* | *5.00E-05* |
| *Alas1* | *aminolevulinic acid synthase 1* | *1.64* | *0.0006* |
| *Alas2* | *aminolevulinic acid synthase 2, erythroid* | *2.12* | *0.0021* |
| *Aldh18a1* | *aldehyde dehydrogenase 18 family, member A1* | *3.17* | *5.00E-05* |
| *Aldh1b1* | *aldehyde dehydrogenase 1 family, member B1* | *4.72* | *5.00E-05* |
| *Aldh3a2* | *aldehyde dehydrogenase family 3, subfamily A2* | *1.80* | *0.00035* |
| *Aldh4a1* | *aldehyde dehydrogenase 4 family, member A1* | *-2.02* | *5.00E-05* |
| *Aldh5a1* | *aldhehyde dehydrogenase family 5, subfamily A1* | *1.89* | *5.00E-05* |
| *Aldh6a1* | *aldehyde dehydrogenase family 6, subfamily A1* | *-1.66* | *0.00065* |
| *Amacr* | *alpha-methylacyl-CoA racemase* | *1.68* | *0.00105* |
| *Apoa1bp* | *apolipoprotein A-I binding protein* | *1.81* | *5.00E-05* |
| *Apool* | *apolipoprotein O-like* | *1.68* | *0.0012* |
| *Arsb* | *arylsulfatase B* | *2.24* | *5.00E-05* |
| *Atp5a1* | *ATP synthase, H+ transporting, mitochondrial F1 complex, alpha subunit 1* | *1.65* | *0.0036* |
| *Atp5b* | *ATP synthase, H+ transporting mitochondrial F1 complex, beta subunit* | *1.86* | *0.0006* |
| *Atp5e* | *ATP synthase, H+ transporting, mitochondrial F1 complex, epsilon subunit* | *1.83* | *5.00E-05* |
| *Atp5f1* | *ATP synthase, H+ transporting, mitochondrial F0 complex, subunit B1* | *1.90* | *5.00E-05* |
| *Atp5g1* | *ATP synthase, H+ transporting, mitochondrial F0 complex, subunit C1* | *2.36* | *5.00E-05* |
| *Atp5g3* | *ATP synthase, H+ transporting, mitochondrial F0 complex, subunit C3* | *1.90* | *5.00E-05* |
| *Atp5h* | *ATP synthase, H+ transporting, mitochondrial F0 complex, subunit D* | *1.63* | *0.0007* |
| *Atp5j* | *ATP synthase, H+ transporting, mitochondrial F0 complex, subunit F* | *2.03* | *5.00E-05* |
| *Atp5j2* | *ATP synthase, H+ transporting, mitochondrial F0 complex, subunit F2* | *2.14* | *5.00E-05* |
| *Atp5k* | *ATP synthase, H+ transporting, mitochondrial F1F0 complex, subunit E* | *2.70* | *5.00E-05* |
| *Atp5l* | *ATP synthase, H+ transporting, mitochondrial F0 complex, subunit G* | *2.11* | *5.00E-05* |
| *Atp5o* | *ATP synthase, H+ transporting, mitochondrial F1 complex, O subunit* | *1.95* | *5.00E-05* |
| *Atp6v1a* | *ATPase, H+ transporting, lysosomal V1 subunit A* | *2.04* | *5.00E-05* |
| *Atpaf1* | *ATP synthase mitochondrial F1 complex assembly factor 1* | *2.27* | *5.00E-05* |
| *Atpif1* | *ATPase inhibitory factor 1* | *1.77* | *0.00045* |
| *Bak1* | *BCL2-antagonist/killer 1* | *1.71* | *0.00775* |
| *Bcat2* | *branched chain aminotransferase 2, mitochondrial* | *-2.34* | *5.00E-05* |
| *Bckdhb* | *branched chain ketoacid dehydrogenase E1, beta polypeptide* | *-1.83* | *0.00045* |
| *Bcl2l1* | *BCL2-like 1* | *2.14* | *5.00E-05* |
| *Bid* | *BH3 interacting domain death agonist* | *4.46* | *5.00E-05* |
| *Bok* | *BCL2-related ovarian killer* | *1.89* | *0.0126* |
| *Bri3bp* | *Bri3 binding protein* | *2.42* | *5.00E-05* |
| *Camk2a* | *calcium/calmodulin-dependent protein kinase II alpha* | *-3.07* | *5.00E-05* |
| *Casp8* | *caspase 8* | *2.43* | *5.00E-05* |
| *Casp9* | *caspase 9* | *2.45* | *5.00E-05* |
| *Casq1* | *calsequestrin 1* | *-2.46* | *5.00E-05* |
| *Cat* | *catalase* | *1.76* | *0.0001* |
| *Cav1* | *caveolin 1, caveolae protein* | *1.93* | *5.00E-05* |
| *Cbr4* | *carbonyl reductase 4* | *1.95* | *5.00E-05* |
| *Ccdc51* | *coiled-coil domain containing 51* | *1.65* | *0.012* |
| *Cd36* | *CD36 antigen* | *2.56* | *5.00E-05* |
| *Cdk1* | *cyclin-dependent kinase 1* | *4.64* | *0.0022* |
| *Cdk5rap1* | *CDK5 regulatory subunit associated protein 1* | *1.80* | *0.00105* |
| *Cds2* | *CDP-diacylglycerol synthase (phosphatidate cytidylyltransferase) 2* | *2.30* | *5.00E-05* |
| *Cerk* | *ceramide kinase* | *1.90* | *0.0002* |
| *Chchd3* | *coiled-coil-helix-coiled-coil-helix domain containing 3* | *1.69* | *0.00015* |
| *Chchd4* | *coiled-coil-helix-coiled-coil-helix domain containing 4* | *2.11* | *5.00E-05* |
| *Chmp2b* | *charged multivesicular body protein 2B* | *1.61* | *0.0025* |
| *Ckmt2* | *creatine kinase, mitochondrial 2* | *3.51* | *5.00E-05* |
| *Clic1* | *chloride intracellular channel 1* | *1.62* | *0.0043* |
| *Clic4* | *chloride intracellular channel 4* | *1.69* | *0.00045* |
| *Clu* | *clusterin* | *-2.60* | *5.00E-05* |
| *Cmc4* | *C-x(9)-C motif containing 4* | *1.74* | *0.02505* |
| *Cmpk2* | *cytidine monophosphate kinase 2, mitochondrial* | *1.83* | *0.00255* |
| *Coasy* | *Coenzyme A synthase* | *2.19* | *5.00E-05* |
| *Comt* | *catechol-O-methyltransferase* | *1.75* | *0.005* |
| *Coq3* | *coenzyme Q3 methyltransferase* | *1.88* | *0.00015* |
| *Coq4* | *coenzyme Q4* | *1.69* | *0.0067* |
| *Coq5* | *coenzyme Q5 methyltransferase* | *1.61* | *0.00105* |
| *Coq6* | *coenzyme Q6 monooxygenase* | *1.62* | *0.002* |
| *Coq7* | *demethyl-Q7* | *1.63* | *0.0008* |
| *Cox4i1* | *cytochrome c oxidase subunit IV isoform 1* | *1.84* | *5.00E-05* |
| *Cox5a* | *cytochrome c oxidase subunit Va* | *1.92* | *5.00E-05* |
| *Cox5b* | *cytochrome c oxidase subunit Vb* | *2.41* | *5.00E-05* |
| *Cox6a1* | *cytochrome c oxidase subunit VIa polypeptide 1* | *2.71* | *5.00E-05* |
| *Cox6b1* | *cytochrome c oxidase, subunit VIb polypeptide 1* | *2.23* | *5.00E-05* |
| *Cox6c* | *cytochrome c oxidase subunit Vic* | *2.28* | *5.00E-05* |
| *Cox7a1* | *cytochrome c oxidase subunit VIIa 1* | *2.00* | *5.00E-05* |
| *Cox7a2* | *cytochrome c oxidase subunit VIIa 2* | *2.02* | *5.00E-05* |
| *Cox7a2l* | *cytochrome c oxidase subunit VIIa polypeptide 2-like* | *1.72* | *0.0001* |
| *Cox7b* | *cytochrome c oxidase subunit VIIb* | *1.99* | *5.00E-05* |
| *Cox7c* | *cytochrome c oxidase subunit VIIc* | *2.56* | *5.00E-05* |
| *Cox8a* | *cytochrome c oxidase subunit VIIIa* | *1.89* | *5.00E-05* |
| *Cpt1a* | *carnitine palmitoyltransferase 1a, liver* | *2.21* | *0.00005* |
| *Cpt1b* | *carnitine palmitoyltransferase 1b, muscle* | *1.59* | *0.00105* |
| *Cpt2* | *carnitine palmitoyltransferase 2* | *1.83* | *5.00E-05* |
| *Cryab* | *crystallin, alpha B* | *4.15* | *5.00E-05* |
| *Ctsb* | *cathepsin B* | *1.73* | *0.00035* |
| *Ctsd* | *cathepsin D* | *1.65* | *0.00045* |
| *Cyb5r2* | *cytochrome b5 reductase 2* | *4.12* | *5.00E-05* |
| *Cyc1* | *cytochrome c-1* | *2.25* | *5.00E-05* |
| *Cycs* | *cytochrome c, somatic* | *2.01* | *5.00E-05* |
| *Cyp1b1* | *cytochrome P450, family 1, subfamily b, polypeptide 1* | *2.89* | *0.0013* |
| *Cyp27a1* | *cytochrome P450, family 27, subfamily a, polypeptide 1* | *-5.02* | *5.00E-05* |
| *Cyp2e1* | *cytochrome P450, family 2, subfamily e, polypeptide 1* | *-2.20* | *5.00E-05* |
| *Dact2* | *dishevelled-binding antagonist of beta-catenin 2* | *-1.72* | *0.0161* |
| *Dcakd* | *dephospho-CoA kinase domain containing* | *1.88* | *0.0002* |
| *Decr1* | *2,4-dienoyl CoA reductase 1, mitochondrial* | *1.85* | *5.00E-05* |
| *Degs1* | *delta(4)-desaturase, sphingolipid 1* | *2.64* | *5.00E-05* |
| *Dgat2* | *diacylglycerol O-acyltransferase 2* | *2.01* | *5.00E-05* |
| *Dhrs4* | *dehydrogenase/reductase member 4* | *1.89* | *5.00E-05* |
| *Disc1* | *disrupted in schizophrenia 1* | *2.03* | *0.0238* |
| *Dlat* | *dihydrolipoamide S-acetyltransferase* | *2.13* | *5.00E-05* |
| *Dlst* | *dihydrolipoamide S-succinyltransferase* | *1.99* | *5.00E-05* |
| *Dna2* | *DNA replication helicase/nuclease 2* | *2.54* | *0.00795* |
| *Dnajc4* | *DnaJ heat shock protein family member C4* | *2.03* | *0.00005* |
| *Dpysl2* | *dihydropyrimidinase-like 2* | *2.04* | *0.00015* |
| *Dusp26* | *dual specificity phosphatase 26* | *-3.46* | *5.00E-05* |
| *Dynll1* | *dynein light chain LC8-type 1* | *1.91* | *5.00E-05* |
| *Ech1* | *enoyl coenzyme A hydratase 1, peroxisomal* | *1.87* | *0.00005* |
| *Echdc2* | *enoyl Coenzyme A hydratase domain containing 2* | *2.06* | *0.0003* |
| *Echs1* | *enoyl Coenzyme A hydratase, short chain, 1, mitochondrial* | *1.78* | *5.00E-05* |
| *Eci1* | *enoyl-Coenzyme A delta isomerase 1* | *2.78* | *5.00E-05* |
| *Elk3* | *ELK3, member of ETS oncogene family* | *5.35* | *0.0016* |
| *Ern1* | *endoplasmic reticulum to nucleus signalling 1* | *3.87* | *5.00E-05* |
| *Etfa* | *electron transferring flavoprotein, alpha polypeptide* | *1.79* | *5.00E-05* |
| *Etfdh* | *electron transferring flavoprotein, dehydrogenase* | *1.85* | *5.00E-05* |
| *Fads1* | *fatty acid desaturase 1* | *1.61* | *0.0047* |
| *Fam110b* | *family with sequence similarity 110, member B* | *2.29* | *0.00035* |
| *Fam213a* | *family with sequence similarity 213, member A* | *1.63* | *0.0027* |
| *Fancg* | *Fanconi anemia, complementation group G* | *2.92* | *0.00015* |
| *Fars2* | *phenylalanine-tRNA synthetase 2* | *1.90* | *5.00E-05* |
| *Fdxr* | *ferredoxin reductase* | *2.35* | *5.00E-05* |
| *Foxred1* | *FAD-dependent oxidoreductase domain containing 1* | *1.82* | *0.0001* |
| *G0s2* | *G0/G1 switch gene 2* | *4.45* | *5.00E-05* |
| *Galc* | *galactosylceramidase* | *2.17* | *0.0001* |
| *Gcat* | *glycine C-acetyltransferase* | *-3.00* | *0.00005* |
| *Gimap3* | *GTPase, IMAP family member 3* | *3.16* | *0.02375* |
| *Gimap5* | *GTPase, IMAP family member 5* | *3.09* | *0.00015* |
| *Gja1* | *gap junction protein, alpha 1* | *2.58* | *5.00E-05* |
| *Glrx* | *glutaredoxin* | *1.92* | *5.00E-05* |
| *Glrx5* | *glutaredoxin 5* | *1.64* | *0.00035* |
| *Glul* | *glutamate-ammonia ligase* | *-1.74* | *0.0001* |
| *Gng5* | *guanine nucleotide binding protein, gamma 5* | *1.64* | *0.0007* |
| *Got2* | *glutamatic-oxaloacetic transaminase 2, mitochondrial* | *2.75* | *5.00E-05* |
| *Gpd1* | *glycerol-3-phosphate dehydrogenase 1* | *-2.00* | *5.00E-05* |
| *Gprc5c* | *G protein-coupled receptor, family C, group 5, member C* | *-2.45* | *5.00E-05* |
| *Gpt2* | *glutamic pyruvate transaminase 2* | *-2.62* | *5.00E-05* |
| *Gpx1* | *glutathione peroxidase 1* | *1.74* | *0.0001* |
| *Grn* | *granulin* | *2.52* | *5.00E-05* |
| *Grpel1* | *GrpE-like 1, mitochondrial* | *1.59* | *0.0046* |
| *Grpel2* | *GrpE-like 2, mitochondrial* | *3.56* | *5.00E-05* |
| *Gsr* | *glutathione reductase* | *1.77* | *0.00015* |
| *Gstk1* | *glutathione S-transferase kappa 1* | *-1.77* | *0.00045* |
| *H6pd* | *hexose-6-phosphate dehydrogenase* | *-1.90* | *5.00E-05* |
| *Hadh* | *hydroxyacyl-Coenzyme A dehydrogenase* | *1.68* | *0.0001* |
| *Hadha* | *hydroxyacyl-Coenzyme A dehydrogenase/3-ketoacyl-Coenzyme A thiolase/enoyl-Coenzyme A hydratase, alpha subunit* | *1.94* | *5.00E-05* |
| *Hadhb* | *hydroxyacyl-Coenzyme A dehydrogenase/3-ketoacyl-Coenzyme A thiolase/enoyl-Coenzyme A hydratase, beta subunit* | *2.21* | *5.00E-05* |
| *Hagh* | *hydroxyacyl glutathione hydrolase* | *2.39* | *5.00E-05* |
| *Hccs* | *holocytochrome c synthetase* | *2.44* | *5.00E-05* |
| *Hcls1* | *hematopoietic cell specific Lyn substrate 1* | *1.99* | *0.0009* |
| *Hdhd3* | *haloacid dehalogenase-like hydrolase domain containing 3* | *2.76* | *0.00425* |
| *Hibch* | *3-hydroxyisobutyryl-Coenzyme A hydrolase* | *1.60* | *0.0028* |
| *Hk1* | *hexokinase 1* | *1.75* | *0.0004* |
| *Hk2* | *hexokinase 2* | *2.12* | *5.00E-05* |
| *Hmgcl* | *3-hydroxy-3-methylglutaryl-Coenzyme A lyase* | *1.69* | *0.0005* |
| *Hs1bp3* | *HCLS1 binding protein 3* | *1.98* | *0.00025* |
| *Hscb* | *HscB iron-sulfur cluster co-chaperone* | *1.81* | *0.00185* |
| *Hsd17b4* | *hydroxysteroid dehydrogenase 4* | *1.76* | *5.00E-05* |
| *Hsdl2* | *hydroxysteroid dehydrogenase like 2* | *1.79* | *5.00E-05* |
| *Hspa1a* | *heat shock protein 1A* | *1.66* | *0.00635* |
| *Idh1* | *isocitrate dehydrogenase 1, soluble* | *1.66* | *0.00105* |
| *Idh2* | *isocitrate dehydrogenase 2, mitochondrial* | *2.41* | *5.00E-05* |
| *Idh3b* | *isocitrate dehydrogenase 3 beta* | *1.60* | *0.00095* |
| *Immp2l* | *IMP2 inner mitochondrial membrane peptidase-like* | *2.00* | *0.00025* |
| *Isca2* | *iron-sulfur cluster assembly 2* | *1.75* | *0.0003* |
| *Isoc2a* | *isochorismatase domain containing 2a* | *-1.66* | *0.00225* |
| *Ivd* | *isovaleryl coenzyme A dehydrogenase* | *-2.30* | *5.00E-05* |
| *Kcnj11* | *potassium inwardly rectifying channel, subfamily J, member 11* | *-1.65* | *0.00045* |
| *Kcnj8* | *potassium inwardly-rectifying channel, subfamily J, member 8* | *1.75* | *0.0004* |
| *Kcnma1* | *potassium large conductance calcium-activated channel, subfamily M, alpha member 1* | *-1.76* | *0.0006* |
| *Kmo* | *kynurenine 3-monooxygenase* | *3.39* | *0.0194* |
| *Lactb2* | *lactamase, beta 2* | *1.76* | *0.0005* |
| *Ldhb* | *lactate dehydrogenase B* | *5.25* | *5.00E-05* |
| *Letm1* | *leucine zipper-EF-hand containing transmembrane protein 1* | *1.60* | *0.0009* |
| *Lipt1* | *lipoyltransferase 1* | *2.51* | *5.00E-05* |
| *Lyrm4* | *LYR motif containing 4* | *2.12* | *0.0001* |
| *Lyrm5* | *LYR motif containing 5* | *1.75* | *0.0001* |
| *Mapk3* | *mitogen-activated protein kinase 3* | *3.79* | *0.00005* |
| *Mapk9* | *mitogen-activated protein kinase 9* | *-1.72* | *0.0006* |
| *Mars2* | *methionine-tRNA synthetase 2* | *1.75* | *0.0027* |
| *Mcl1* | *myeloid cell leukemia sequence 1* | *1.69* | *0.0001* |
| *Mcu* | *mitochondrial calcium uniporter* | *1.64* | *0.0008* |
| *Mdh1* | *malate dehydrogenase 1, NAD* | *2.57* | *5.00E-05* |
| *Mdh2* | *malate dehydrogenase 2, NAD* | *1.90* | *5.00E-05* |
| *Me1* | *malic enzyme 1, NADP(+)-dependent, cytosolic* | *-2.68* | *5.00E-05* |
| *Me2* | *malic enzyme 2, NAD(+)-dependent, mitochondrial* | *2.28* | *0.0002* |
| *Minos1* | *mitochondrial inner membrane organizing system 1* | *1.80* | *5.00E-05* |
| *Mmab* | *methylmalonic aciduria cblB type homolog* | *-2.00* | *5.00E-05* |
| *Mmachc* | *methylmalonic aciduria cblC type, with homocystinuria* | *1.65* | *0.0008* |
| *Mpst* | *mercaptopyruvate sulfurtransferase* | *-2.37* | *5.00E-05* |
| *Mrm1* | *mitochondrial rRNA methyltransferase 1* | *1.90* | *0.0001* |
| *Mrpl10* | *mitochondrial ribosomal protein L10* | *1.60* | *0.0015* |
| *Mrpl12* | *mitochondrial ribosomal protein L12* | *1.88* | *5.00E-05* |
| *Mrpl13* | *mitochondrial ribosomal protein L13* | *1.59* | *0.00165* |
| *Mrpl18* | *mitochondrial ribosomal protein L18* | *1.63* | *0.00085* |
| *Mrpl19* | *mitochondrial ribosomal protein L19* | *1.67* | *0.0024* |
| *Mrpl28* | *mitochondrial ribosomal protein L28* | *1.79* | *0.0002* |
| *Mrpl3* | *mitochondrial ribosomal protein L3* | *3.20* | *5.00E-05* |
| *Mrpl30* | *mitochondrial ribosomal protein L30* | *1.69* | *0.00025* |
| *Mrpl37* | *mitochondrial ribosomal protein L37* | *1.62* | *0.0005* |
| *Mrpl4* | *mitochondrial ribosomal protein L4* | *1.81* | *5.00E-05* |
| *Mrpl41* | *mitochondrial ribosomal protein L41* | *2.36* | *0.00805* |
| *Mrpl42* | *mitochondrial ribosomal protein L42* | *1.97* | *5.00E-05* |
| *Mrpl45* | *mitochondrial ribosomal protein L45* | *2.01* | *5.00E-05* |
| *Mrpl46* | *mitochondrial ribosomal protein L46* | *1.89* | *5.00E-05* |
| *Mrpl47* | *mitochondrial ribosomal protein L47* | *2.12* | *0.00425* |
| *Mrpl50* | *mitochondrial ribosomal protein L50* | *1.78* | *0.0001* |
| *Mrpl51* | *mitochondrial ribosomal protein L51* | *1.74* | *5.00E-05* |
| *Mrpl55* | *mitochondrial ribosomal protein L55* | *1.87* | *5.00E-05* |
| *Mrpl57* | *mitochondrial ribosomal protein L57* | *1.83* | *5.00E-05* |
| *Mrps12* | *mitochondrial ribosomal protein S12* | *1.67* | *0.00075* |
| *Mrps16* | *mitochondrial ribosomal protein S16* | *1.60* | *0.003* |
| *Mrps18a* | *mitochondrial ribosomal protein S18A* | *2.25* | *5.00E-05* |
| *Mrps2* | *mitochondrial ribosomal protein S2* | *2.15* | *5.00E-05* |
| *Mrps24* | *mitochondrial ribosomal protein S24* | *1.74* | *0.0002* |
| *Mrps25* | *mitochondrial ribosomal protein S25* | *1.61* | *0.00395* |
| *Mrps35* | *mitochondrial ribosomal protein S35* | *1.79* | *0.0005* |
| *Mrps36* | *mitochondrial ribosomal protein S36* | *2.20* | *5.00E-05* |
| *Mrps9* | *mitochondrial ribosomal protein S9* | *1.86* | *5.00E-05* |
| *Mrrf* | *mitochondrial ribosome recycling factor* | *1.61* | *0.04385* |
| *Msrb2* | *methionine sulfoxide reductase B2* | *2.34* | *5.00E-05* |
| *Msrb3* | *methionine sulfoxide reductase B3* | *-1.94* | *5.00E-05* |
| *Msto1* | *misato 1, mitochondrial distribution and morphology regulator* | *2.04* | *0.01175* |
| *Mtch2* | *mitochondrial carrier 2* | *1.72* | *0.0001* |
| *Mtcp1* | *mature T cell proliferation 1* | *1.74* | *0.02505* |
| *Mterf2* | *mitochondrial transcription termination factor 2* | *1.70* | *0.0041* |
| *Mterf4* | *mitochondrial transcription termination factor 4* | *1.62* | *0.0378* |
| *Mtfmt* | *mitochondrial methionyl-tRNA formyltransferase* | *1.68* | *0.01295* |
| *Mtg1* | *mitochondrial ribosome-associated GTPase 1* | *1.89* | *0.0001* |
| *Mthfd1l* | *methylenetetrahydrofolate dehydrogenase 1-like* | *1.95* | *0.04745* |
| *Mthfd2* | *methylenetetrahydrofolate dehydrogenase, methenyltetrahydrofolate cyclohydrolase* | *8.27* | *5.00E-05* |
| *Mtif3* | *mitochondrial translational initiation factor 3* | *1.68* | *0.0175* |
| *Mtor* | *mechanistic target of rapamycin* | *3.28* | *5.00E-05* |
| *Mtrf1l* | *mitochondrial translational release factor 1-like* | *1.66* | *0.00995* |
| *Mtx2* | *metaxin 2* | *2.77* | *5.00E-05* |
| *Mul1* | *mitochondrial ubiquitin ligase activator of NFKB 1* | *1.64* | *0.00085* |
| *Mutyh* | *mutY DNA glycosylase* | *2.19* | *0.0064* |
| *Myc* | *myelocytomatosis oncogene* | *-1.67* | *0.0189* |
| *Myo19* | *myosin XIX* | *2.26* | *0.00795* |
| *Myoc* | *myocilin* | *-2.32* | *5.00E-05* |
| *Ndrg4* | *N-myc downstream regulated gene 4* | *2.58* | *5.00E-05* |
| *Ndufa1* | *NADH dehydrogenase 1 alpha subcomplex, 1* | *2.02* | *5.00E-05* |
| *Ndufa10* | *NADH dehydrogenase 1 alpha subcomplex 10* | *2.10* | *5.00E-05* |
| *Ndufa11* | *NADH dehydrogenase 1 alpha subcomplex 11* | *1.81* | *5.00E-05* |
| *Ndufa13* | *NADH dehydrogenase 1 alpha subcomplex, 13* | *1.90* | *5.00E-05* |
| *Ndufa3* | *NADH dehydrogenase 1 alpha subcomplex, 3* | *1.61* | *0.00155* |
| *Ndufa4* | *NADH dehydrogenase 1 alpha subcomplex, 4* | *2.19* | *5.00E-05* |
| *Ndufa5* | *NADH dehydrogenase 1 alpha subcomplex, 5* | *1.97* | *5.00E-05* |
| *Ndufa6* | *NADH dehydrogenase 1 alpha subcomplex, 6* | *1.61* | *0.00085* |
| *Ndufa8* | *NADH dehydrogenase 1 alpha subcomplex, 8* | *1.86* | *5.00E-05* |
| *Ndufa9* | *NADH dehydrogenase 1 alpha subcomplex, 9* | *1.78* | *5.00E-05* |
| *Ndufab1* | *NADH dehydrogenase 1, alpha/beta subcomplex, 1* | *2.05* | *5.00E-05* |
| *Ndufaf1* | *NADH dehydrogenase 1 alpha subcomplex, assembly factor 1* | *1.69* | *0.00035* |
| *Ndufaf2* | *NADH dehydrogenase 1 alpha subcomplex, assembly factor 2* | *1.60* | *0.01205* |
| *Ndufb10* | *NADH dehydrogenase 1 beta subcomplex, 10* | *1.75* | *5.00E-05* |
| *Ndufb11* | *NADH dehydrogenase 1 beta subcomplex, 11* | *1.67* | *0.00045* |
| *Ndufb2* | *NADH dehydrogenase 1 beta subcomplex, 2* | *1.59* | *0.00115* |
| *Ndufb3* | *NADH dehydrogenase 1 beta subcomplex, 3* | *1.66* | *0.0003* |
| *Ndufb5* | *NADH dehydrogenase 1 beta subcomplex, 5* | *2.07* | *5.00E-05* |
| *Ndufb6* | *NADH dehydrogenase 1 beta subcomplex, 6* | *1.72* | *0.0001* |
| *Ndufb7* | *NADH dehydrogenase 1 beta subcomplex, 7* | *1.67* | *0.0003* |
| *Ndufb8* | *NADH dehydrogenase 1 beta subcomplex, 8* | *1.76* | *5.00E-05* |
| *Ndufs3* | *NADH dehydrogenase Fe-S protein 3* | *2.12* | *5.00E-05* |
| *Ndufs4* | *NADH dehydrogenase Fe-S protein 4* | *1.63* | *0.00055* |
| *Ndufs5* | *NADH dehydrogenase Fe-S protein 5* | *1.59* | *0.0008* |
| *Ndufs8* | *NADH dehydrogenase Fe-S protein 8* | *1.66* | *0.0001* |
| *Ndufv1* | *NADH dehydrogenase flavoprotein 1* | *1.89* | *5.00E-05* |
| *Ndufv2* | *NADH dehydrogenase flavoprotein 2* | *1.69* | *0.0001* |
| *Nipsnap3b* | *nipsnap homolog 3B* | *2.02* | *0.00415* |
| *Nln* | *neurolysin* | *2.60* | *5.00E-05* |
| *Nme1* | *NME/NM23 nucleoside diphosphate kinase 1* | *2.27* | *5.00E-05* |
| *Nme2* | *NME/NM23 nucleoside diphosphate kinase 2* | *1.70* | *5.00E-05* |
| *Nme4* | *NME/NM23 nucleoside diphosphate kinase 4* | *2.37* | *0.00215* |
| *Nnt* | *nicotinamide nucleotide transhydrogenase* | *5.50* | *5.00E-05* |
| *Nsun4* | *NOL1/NOP2/Sun domain family, member 4* | *2.26* | *5.00E-05* |
| *Nt5c* | *5',3'-nucleotidase, cytosolic* | *2.15* | *0.0008* |
| *Nubpl* | *nucleotide binding protein-like* | *1.67* | *0.00855* |
| *Nudt19* | *nudix-type motif 19* | *1.95* | *5.00E-05* |
| *Nudt8* | *nudix-type motif 8* | *2.64* | *5.00E-05* |
| *Oas1a* | *2'-5' oligoadenylate synthetase 1A* | *2.17* | *0.00735* |
| *Oas2* | *2'-5' oligoadenylate synthetase 2* | *1.96* | *0.0049* |
| *Ogdh* | *oxoglutarate dehydrogenase* | *2.21* | *5.00E-05* |
| *Ogdhl* | *oxoglutarate dehydrogenase-like* | *10.97* | *5.00E-05* |
| *Oxct1* | *3-oxoacid CoA transferase 1* | *1.60* | *0.001* |
| *P2ry1* | *purinergic receptor P2Y, G-protein coupled 1* | *1.93* | *0.00015* |
| *Park7* | *Parkinson disease 7* | *-3.82* | *5.00E-05* |
| *Parl* | *presenilin associated, rhomboid-like* | *1.73* | *0.0002* |
| *Pck2* | *phosphoenolpyruvate carboxykinase 2* | *2.34* | *5.00E-05* |
| *Pcx* | *pyruvate carboxylase* | *-2.39* | *5.00E-05* |
| *Pdha1* | *pyruvate dehydrogenase E1 alpha 1* | *2.06* | *5.00E-05* |
| *Pdhb* | *pyruvate dehydrogenase (lipoamide) beta* | *3.44* | *5.00E-05* |
| *Pdk3* | *pyruvate dehydrogenase kinase, isoenzyme 3* | *2.54* | *0.0043* |
| *Pdss1* | *prenyl (solanesyl) diphosphate synthase, subunit 1* | *1.92* | *0.0163* |
| *Pdss2* | *prenyl (solanesyl) diphosphate synthase, subunit 2* | *1.66* | *0.00105* |
| *Perp* | *PERP, TP53 apoptosis effector* | *5.24* | *5.00E-05* |
| *Phb* | *prohibitin* | *1.68* | *0.0001* |
| *Phb2* | *prohibitin 2* | *1.78* | *5.00E-05* |
| *Pink1* | *PTEN induced putative kinase 1* | *-1.64* | *0.00055* |
| *Pisd* | *phosphatidylserine decarboxylase* | *2.96* | *5.00E-05* |
| *Plekhf1* | *pleckstrin homology domain containing, family F member 1* | *2.07* | *5.00E-05* |
| *Pln* | *phospholamban* | *18.12* | *0.00295* |
| *Plscr3* | *phospholipid scramblase 3* | *1.73* | *0.00265* |
| *Ppif* | *peptidylprolyl isomerase F* | *2.90* | *5.00E-05* |
| *Ppl* | *periplakin* | *-5.61* | *5.00E-05* |
| *Ppm1k* | *protein phosphatase 1K* | *-1.85* | *0.0031* |
| *Prdx2* | *peroxiredoxin 2* | *2.37* | *5.00E-05* |
| *Prdx3* | *peroxiredoxin 3* | *1.61* | *0.0008* |
| *Prdx6* | *peroxiredoxin 6* | *2.24* | *5.00E-05* |
| *Prelid1* | *PRELI domain containing 1* | *1.77* | *0.00025* |
| *Prelid2* | *PRELI domain containing 2* | *7.16* | *0.0099* |
| *Prosc* | *proline synthetase co-transcribed* | *2.54* | *5.00E-05* |
| *Prr5l* | *proline rich 5 like* | *2.34* | *0.0175* |
| *Ptcd2* | *pentatricopeptide repeat domain 2* | *1.62* | *0.00185* |
| *Pycard* | *PYD and CARD domain containing* | *13.81* | *5.00E-05* |
| *Qrsl1* | *glutaminyl-tRNA synthase-like 1* | *1.70* | *0.00065* |
| *Rab11fip5* | *RAB11 family interacting protein 5* | *1.79* | *5.00E-05* |
| *Rai14* | *retinoic acid induced 14* | *2.88* | *5.00E-05* |
| *Rdh13* | *retinol dehydrogenase 13* | *1.79* | *0.0001* |
| *Ripk3* | *receptor-interacting serine-threonine kinase 3* | *2.13* | *0.012* |
| *Rmdn3* | *regulator of microtubule dynamics 3* | *1.61* | *0.01635* |
| *Rmnd1* | *required for meiotic nuclear division 1 homolog* | *1.80* | *0.0002* |
| *Rnf144b* | *ring finger protein 144B* | *1.64* | *0.0007* |
| *Rsad2* | *radical S-adenosyl methionine domain containing 2* | *3.08* | *5.00E-05* |
| *Rtn4ip1* | *reticulon 4 interacting protein 1* | *1.89* | *5.00E-05* |
| *Sardh* | *sarcosine dehydrogenase* | *1.71* | *0.0178* |
| *Sco1* | *SCO1 cytochrome c oxidase assembly protein* | *2.02* | *0.00155* |
| *Scp2* | *sterol carrier protein 2, liver* | *1.62* | *0.0007* |
| *Sdhaf2* | *succinate dehydrogenase complex assembly factor 2* | *1.68* | *0.00565* |
| *Sdhaf4* | *succinate dehydrogenase complex assembly factor 4* | *2.07* | *5.00E-05* |
| *Sdhb* | *succinate dehydrogenase complex, subunit B, iron sulfur* | *1.87* | *5.00E-05* |
| *Sdhc* | *succinate dehydrogenase complex, subunit C, integral membrane protein* | *1.99* | *5.00E-05* |
| *Sdhd* | *succinate dehydrogenase complex, subunit D, integral membrane protein* | *2.04* | *5.00E-05* |
| *Sdsl* | *serine dehydratase-like* | *3.64* | *0.03065* |
| *Sfxn1* | *sideroflexin 1* | *1.90* | *0.0039* |
| *Sfxn5* | *sideroflexin 5* | *1.84* | *0.0024* |
| *Sh3glb1* | *SH3-domain GRB2-like B1* | *2.18* | *5.00E-05* |
| *Shc1* | *src homology 2 domain-containing transforming protein C1* | *1.60* | *0.0023* |
| *Shmt2* | *serine hydroxymethyltransferase 2* | *1.84* | *0.0006* |
| *Sirt3* | *sirtuin 3* | *2.65* | *5.00E-05* |
| *Sirt5* | *sirtuin 5* | *1.87* | *0.00045* |
| *Slc16a1* | *solute carrier family 16, member 1* | *2.67* | *5.00E-05* |
| *Slc22a4* | *solute carrier family 22, member 4* | *2.55* | *0.0001* |
| *Slc25a10* | *solute carrier family 25, member 10* | *1.74* | *0.0024* |
| *Slc25a11* | *solute carrier family 25, member 11* | *1.80* | *5.00E-05* |
| *Slc25a12* | *solute carrier family 25, member 12* | *1.71* | *0.00015* |
| *Slc25a13* | *solute carrier family 25, member 13* | *4.12* | *0.0001* |
| *Slc25a20* | *solute carrier family 25, member 20(Slc25a20)* | *1.71* | *0.00025* |
| *Slc25a22* | *solute carrier family 25, member 22* | *2.22* | *5.00E-05* |
| *Slc25a24* | *solute carrier family 25 , member 24* | *3.62* | *5.00E-05* |
| *Slc25a3* | *solute carrier family 25, member 3* | *2.00* | *5.00E-05* |
| *Slc25a34* | *solute carrier family 25, member 34* | *4.22* | *0.00005* |
| *Slc25a36* | *solute carrier family 25, member 36* | *1.77* | *0.0006* |
| *Slc25a39* | *solute carrier family 25, member 39* | *1.96* | *0.00875* |
| *Slc25a4* | *solute carrier family 25, member 4* | *2.46* | *5.00E-05* |
| *Slc25a45* | *solute carrier family 25, member 45* | *2.39* | *5.00E-05* |
| *Slc25a5* | *solute carrier family 25, member 5* | *2.99* | *0.01225* |
| *Slc8a1* | *solute carrier family 8, member 1* | *2.00* | *5.00E-05* |
| *Slc8b1* | *solute carrier family 8, member B1* | *1.76* | *0.00015* |
| *Slirp* | *SRA stem-loop interacting RNA binding protein* | *4.29* | *5.00E-05* |
| *Slmo2* | *slowmo homolog 2* | *-3.20* | *0.02345* |
| *Smim4* | *small integral membrane protein 4* | *-2.09* | *5.00E-05* |
| *Smurf1* | *SMAD specific E3 ubiquitin protein ligase 1* | *1.88* | *5.00E-05* |
| *Snn* | *stannin* | *-7.55* | *0.00485* |
| *Sod2* | *superoxide dismutase 2, mitochondrial* | *-3.62* | *5.00E-05* |
| *Sord* | *sorbitol dehydrogenase* | *3.10* | *5.00E-05* |
| *Sptlc2* | *serine palmitoyltransferase, long chain base subunit 2* | *1.75* | *0.0018* |
| *Sqrdl* | *sulfide quinone reductase-like* | *-1.82* | *0.0005* |
| *Stard4* | *StAR-related lipid transfer domain containing 4* | *1.96* | *0.0028* |
| *Stom* | *stomatin* | *4.33* | *5.00E-05* |
| *Stxbp1* | *syntaxin binding protein 1* | *1.97* | *0.00015* |
| *Supv3l1* | *suppressor of var1, 3-like 1* | *1.73* | *0.00115* |
| *Sybu* | *syntabulin* | *3.31* | *5.00E-05* |
| *Tango2* | *transport and golgi organization 2* | *2.40* | *5.00E-05* |
| *Tap1* | *transporter 1, ATP-binding cassette, sub-family B* | *1.68* | *0.0066* |
| *Tbrg4* | *transforming growth factor beta regulated gene 4* | *1.78* | *5.00E-05* |
| *Tcirg1* | *T cell, immune regulator 1, ATPase, H+ transporting, lysosomal V0 protein A3* | *1.88* | *0.00065* |
| *Tdrkh* | *tudor and KH domain containing protein* | *-3.10* | *0.0024* |
| *Tefm* | *transcription elongation factor, mitochondrial* | *1.67* | *0.00315* |
| *Timm10* | *translocase of inner mitochondrial membrane 10* | *1.60* | *0.0047* |
| *Timm10b* | *translocase of inner mitochondrial membrane 10B* | *1.68* | *0.0129* |
| *Timm8a1* | *translocase of inner mitochondrial membrane 8A1* | *1.91* | *0.00035* |
| *Timm8b* | *translocase of inner mitochondrial membrane 8B* | *1.68* | *0.0006* |
| *Tmem126a* | *transmembrane protein 126A* | *1.80* | *5.00E-05* |
| *Tmem173* | *transmembrane protein 173* | *3.13* | *5.00E-05* |
| *Tmem65* | *transmembrane protein 65* | *1.74* | *0.00015* |
| *Tmem70* | *transmembrane protein 70* | *2.12* | *5.00E-05* |
| *Tmtc1* | *transmembrane and tetratricopeptide repeat containing 1* | *-2.25* | *5.00E-05* |
| *Tnfrsf1a* | *tumor necrosis factor receptor superfamily, member 1a* | *1.86* | *0.00015* |
| *Tomm22* | *translocase of outer mitochondrial membrane 22 homolog* | *1.63* | *0.0012* |
| *Trmt2b* | *TRM2 tRNA methyltransferase 2B* | *2.69* | *5.00E-05* |
| *Trmu* | *tRNA 5-methylaminomethyl-2-thiouridylate methyltransferase* | *1.82* | *0.0027* |
| *Trp53* | *transformation related protein 53* | *1.87* | *0.0001* |
| *Tsfm* | *Ts translation elongation factor, mitochondrial* | *1.81* | *0.00025* |
| *Tspo* | *translocator protein* | *1.99* | *5.00E-05* |
| *Tst* | *thiosulfate sulfurtransferase, mitochondrial* | *-2.75* | *5.00E-05* |
| *Tufm* | *Tu translation elongation factor, mitochondrial* | *1.99* | *5.00E-05* |
| *Txn1* | *thioredoxin 1* | *1.68* | *0.00035* |
| *Txn2* | *thioredoxin 2* | *1.68* | *0.0002* |
| *Uaca* | *uveal autoantigen with coiled-coil domains and ankyrin repeats* | *-1.87* | *5.00E-05* |
| *Ucp2* | *uncoupling protein 2* | *2.13* | *5.00E-05* |
| *Ung* | *uracil DNA glycosylase* | *4.74* | *5.00E-05* |
| *Uqcr10* | *ubiquinol-cytochrome c reductase, complex III subunit X* | *2.18* | *5.00E-05* |
| *Uqcr11* | *ubiquinol-cytochrome c reductase, complex III subunit XI* | *1.81* | *5.00E-05* |
| *Uqcrb* | *ubiquinol-cytochrome c reductase binding protein* | *1.93* | *5.00E-05* |
| *Uqcrc1* | *ubiquinol-cytochrome c reductase core protein 1* | *1.69* | *5.00E-05* |
| *Uqcrc2* | *ubiquinol cytochrome c reductase core protein 2* | *1.86* | *5.00E-05* |
| *Uqcrfs1* | *ubiquinol-cytochrome c reductase, Rieske iron-sulfur polypeptide 1* | *2.18* | *5.00E-05* |
| *Uqcrq* | *ubiquinol-cytochrome c reductase, complex III subunit VII* | *2.51* | *5.00E-05* |
| *Usmg5* | *upregulated during skeletal muscle growth 5* | *2.37* | *5.00E-05* |
| *Usp30* | *ubiquitin specific peptidase 30* | *1.59* | *0.0068* |
| *Vat1* | *vesicle amine transport 1* | *1.68* | *0.0007* |
| *Vps13c* | *vacuolar protein sorting 13C* | *2.01* | *0.00085* |
| *Vps25* | *vacuolar protein sorting 25* | *1.71* | *0.0011* |
| *Vwa8* | *von Willebrand factor A domain containing 8* | *1.66* | *0.0006* |
| *Wdr81* | *WD repeat domain 81* | *1.72* | *0.00275* |
| *Xaf1* | *XIAP associated factor 1* | *2.81* | *0.0002* |
| *Yars2* | *tyrosyl-tRNA synthetase 2* | *1.64* | *0.003* |
| *Ywhaz* | *tyrosine 3-monooxygenase/tryptophan 5-monooxygenase activation protein, zeta polypeptide* | *1.65* | *0.0006* |
| *Zfhx3* | *zinc finger homeobox 3* | *-1.82* | *0.00385* |
| *1700021F05Rik* | *RIKEN cDNA 1700021F05 gene* | *1.84* | *5.00E-05* |
| *2010107E04Rik* | *RIKEN cDNA 2010107E04 gene* | *2.16* | *5.00E-05* |
| *2310061I04Rik* | *RIKEN cDNA 2310061I04 gene* | *1.65* | *0.00285* |
| *2410015M20Rik* | *RIKEN cDNA 2410015M20 gene* | *2.42* | *5.00E-05* |
| *8430408G22Rik* | *RIKEN cDNA 8430408G22 gene* | *3.00* | *0.0001* |
