# Supplementary material for: AMPK-dependent and -independent coordination of mitochondrial function and muscle fiber type by FNIP1
Source: PLoS Genet. 2021 Mar 29;17(3):e1009488. doi: 10.1371/journal.pgen.1009488 (PMC8031738; doi:10.1371/journal.pgen.1009488)
Supplement: S3 Table — (DOCX) [file pgen.1009488.s012.docx]

**S3 Table. sgRNA for *Fnip1* knockout mice generation**

|  | ***Forward*** | ***Reverse*** |
| --- | --- | --- |
| *sgRNA-1* | *5'-TAGGGAATAGGAAGAATAATAG* | *5'-AAACCTATTATTCTTCCTATTC* |
| *sgRNA-2* | *5'-TAGGTCTTGAATTCATCAATC* | *5'-AAACGATTGATGAATTCAAGA* |
| *sgRNA-3* | *5'-TAGGCTGCTTGGAAATATAGGT* | *5'-AAACACCTATATTTCCAAGCAG* |
| *sgRNA-4* | *5'-TAGGGTATAACCTTTCTATTATT* | *5'-AAACAATAATAGAAAGGTTATAC* |
