# Supplementary material for: AMPK-dependent and -independent coordination of mitochondrial function and muscle fiber type by FNIP1
Source: PLoS Genet. 2021 Mar 29;17(3):e1009488. doi: 10.1371/journal.pgen.1009488 (PMC8031738; doi:10.1371/journal.pgen.1009488)
Supplement: S4 Table — (DOCX) [file pgen.1009488.s013.docx]

**S4 Table. RT-PCR primers**

| ***Mouse Gene*** | ***Forward*** | ***Reverse*** |
| --- | --- | --- |
| *36b4* | *5’-ATCCCTGACGCACCGCCGTGA* | *5’-TGCATCTGCTTGGAGCCCACGT* |
| *Fnip1* | *5'-* *AGTAATGGGCTGCTTGGAAA* | *5'-* *CAAAGAAAGAGGCACTCCTGA* |
| *Fnip2* | *5'-TTGACTCCAGAGCCGTTCA* | *5'-GGAACATCCTCTGCTTTCTGA* |
| *Flcn* | *5'-CTATCCCTGCCCATGTTCTG* | *5'-GTGGAGGTTTGAGCGAGT* |
| *Ldha* | *5'-* *TGCCTACGAGGTGATCAAGCT* | *5'-* *GCACCCGCCTAAGGTTCTTC* |
| *Ldhb* | *5'-* *AGTCTCCCGTGCATCCTCAA* | *5'-* *AGGGTGTCCGCACTCTTCCT* |
| *Myh7* | *5'-GCCAACTATGCTGGAGCTGATGCCC* | *5'-GGTGCGTGGAGCGCAAGTTTGTCATAAG* |
| *Myh2* | *5'-GGCACAAACTGCTGAAGCAGAGGC* | *5'-GGTGCTCCTGAGGTTGGTCATCAGC* |
| *Myh1* | *5'-GGCAGCAGCAGCTGCGGAAGCAGA GTCTGG* | *5'-GAGTGCTCCTCAGATTGGTCATTAGC* |
| *Myh4* | *5'-GAGCTACTGGATGCCAGTGAGCGC* | *5'-CTGGACGATGTCTTCCATCTCTCC* |
| *Tnni1* | *5'-TGAAGCCAAATGCCTCCACAACAC* | *5'-ACACCTTGTGCTTAGAGCCCAGTA* |
| *Tnnt1* | *5'-TGGATCCACCAGCTGGAATCAGAA* | *5'-GCTGATGCGGTTGTAGAGCACATT* |
| *Tnnc1* | *5'-AGCTCATGAAGGACGGTGACAAGA* | *5'-AACCGTGCAAGACCAGCATCTACT* |
| *Tnni2* | *5'-AGCAGCAAGGAGCTGGAAGA* | *5'-ATGGCGTCGGCAGACATAC* |
| *Tnnc2* | *5'-CCATCATCGAGGAGGTGGAC* | *5'-CTTCCCCTTCGCATCCTCTT* |
| *Tnnt3* | *5'-AACTGGAGACTGACAAATTCGAGT* | *5'-GCTGTGCTTCTGGGTTTGGT* |
| *mt-Nd1* | *5’-CCCATTCGCGTTATCTT* | *5’-AAGTTGATCGTAACGGAAGC* |
| *Lpl* | *5’-GATGGACGGTAAGAGTGATTC* | *5’-ATCCAAGGGTAGCAGACAGGT* |
