# Supplementary material for: AMPK-dependent and -independent coordination of mitochondrial function and muscle fiber type by FNIP1
Source: PLoS Genet. 2021 Mar 29;17(3):e1009488. doi: 10.1371/journal.pgen.1009488 (PMC8031738; doi:10.1371/journal.pgen.1009488)
Supplement: Attachment — Submitted filename: PGENETICS-D-20-01073R1_Itemized Response to Reviewers.pdf [file pgen.1009488.s015.pdf]

Itemized Responses to Reviewer #1

"Reviewer #1: In this massive work, Xiao and colleagues elegantly dissected the role of FNIP1 in the control of mitochondrial biogenesis and muscle fibres switching by using a series of genetically modified mouse and cellular models. Intriguingly, the authors found that while the effect on mitochondrial biogenesis is unsurprisingly mediated by the AMPK/PGC1alpha pathway, the effect on fibres type does not involve AMPK/PGC1alpha, although the mechanism of the latter is not further investigated. Importantly, the authors also demostarte that mTORC1 signalling is not involved in FNIP1-dependent activities."

We wish to thank the Reviewer for the critical and constructive review.

"The paper is convincing and the conclusions supported by the data."

Thank you for the positive comment.

"However, there are some comments I would like the authors to consider."

1. *"the title is somehow misleading as it does not mention what is, in my opinion, the most relevant finding, i.e. the fact that AMPK is not involved in the determination of the fibre type by FNIP1"*

We thank the reviewer and we have made a revision of the manuscript title to make mention of the both AMPK-dependent and -independent coordination of mitochondrial function and muscle fiber type by FNIP1.

2. *"in both abstract and authors' summary, the authors stress the fact that FNIP1 can be a novel target for the therapy of a number of diseases. I find this statement generic and completely out of the main subject of the paper, and suggest the authors to remove the sentences."*

We have removed those sentences in the abstract and authors' summary, thanks.

3. *"in figure S2C FLNC and FNIP2 are shown, but no explanation of what they are is given in the main text, although it is present in the Discussion. However, I suggest to move the explanation to the main text for clarity. In particular, it is unclear what FNIP2 is"*

We thank the reviewer. Folliculin (FLCN) and FNIP1 bind to each other (Baba et al., Proc Natl Acad Sci U S A., 2006, 103, 15552-15557), and FNIP2 is a close homolog of FNIP1. As suggested, FLCN and FNIP2 have been explained in the main text (page 5, line 91, page 7, line 138-139).

4. *"Figure 1F, sowing the results from the TEM studies should be properly quantified, and the quality of the images improved as it is difficult to clearly distinguish the mitochondrial ultrastructure."*

We have replaced the original Fig 1F with new TEM images and quantified the mitochondrial area per muscle fiber area as suggested. The data now indicate a clear significant increase in percentage of mitochondrial area in the *Fnip1*^{KO} muscles as compared to WT controls or *Fnip1*^{TgKO} muscles (revised Fig

1F and 1G), which was consistent with mitochondrial DNA measurements (revised Fig 1H). Notably, the WT and *Fnip1*^{TgKO} mice have an ordered array of small intermyofibrillar mitochondria with dense mitochondrial matrix along the Z-lines of the sarcomeres, whereas the *Fnip1*^{KO} mice have a larger shape and less dense mitochondria, which displaced the myofibrils (revised Fig 1F). These results could suggest that FNIP1 not only regulates mitochondria quantity, but also regulate mitochondrial quality. Indeed, during the review of this manuscript, a study was published reporting that FNIP1 regulates mitochondrial redox homeostasis (Manford et al., Cell, 2020, 183, 46-61.e21). It is also possible that the altered mitochondrial morphology is due to the changes in mitochondrial location (e.g. the proximity to the myofibrils triads where calcium is released from SR). This information has been added as revised Fig 1F and 1G (page 9, line 179-183) and addressed in the revised Discussion (page 20, line 434-439).

5. *“In the expression profile 27/34 genes upregulated in the FNIP1 KO muscles are from respiratory complex I, but in the western blot in Figure 2A all the complexes are increased. Are the corresponding transcripts also increased?”*

We have found that a broad array of mitochondrial genes was up-regulated in *Fnip1*^{KO} muscle (Fig 1D and original S1 Table). The regulated genes (a total of 455 genes) were displayed in the original S1 Table (now revised S2 Table), and the mRNA levels of *Ndufb8*, *Uqcrc2*, *Cox4i1* and *Atp5a1* (Western blot in Fig 2A) were all induced in *Fnip1*^{KO} muscle. We have now also added a heat map showing the up-regulated subunits from the mitochondrial respiratory complexes in *Fnip1*^{KO} muscle (revised S3C Fig).

6. *“why are myoglobin and cytochrome c upregulated in FNIP1 KO (Figure 2B)? No comment or discussion is made on this point.”*

Muscle oxidative metabolism biomarkers such as myoglobin and cytochrome c are known PGC-1α targets (Lin et al., Nature, 2002, 418, 797-801; Schreiber et al., Proc Natl Acad Sci U S A., 2004, 101, 6472-6477). We found that the induced expression of myoglobin and cytochrome c protein paralleled the activation of AMPK/PGC-1α signaling in *Fnip1*^{KO} muscle, and knocking out *Ampk*$\alpha 1/\alpha 2$ or PGC-1α blunted the effect of *Fnip1*^{KO} on the expression of myoglobin and cytochrome c protein (original Fig 4E, 5B, and 7D). We have added a sentence to the text to make this point clearer (page 10, line 204).

7. *“in Figure 2C and 2D the authors show a switch in LDH isoforms, however it is unclear if this impacts on the blood lactate levels.”*

The LDHB/LDHA isoenzyme ratio is increased in *Fnip1*^{KO} muscle. These results were of interest. We have conducted additional blood lactate measurement. As predicted, we have found that blood lactate levels were reduced in *Fnip1*^{KO} mice compared to the WT controls, and blood lactate levels from *Fnip1*^{TgKO} mice were increased to a level similar to WT controls. These new results were added in the revised Fig 2E (page 10, line 212-213).

8. *“Page 11 line 233 Fnip1KO induced... should be changed to "increased"”*

This has been corrected. Thanks!

Itemized Responses to Reviewer #2

We sincerely appreciate the Reviewer's critical and constructive review of our manuscript.

Major comments:

> "*Reviewer #2: This paper does not advance the field. The effect of FNIP1 KO in skeletal muscle has been published in 2015 by Reyes et al. The extra data showing that the fiber typ switch is not dependent on AMPK and PGC-1a is not conclusive. It is against Hasumi et al, 2012, who show convincingly that the red muscle fibre phenotype is reversed upon PGC1a KO.*"

We appreciate this important point and have discussed the significance of our study relative to the documented studies the Reviewer mentioned in the revised Discussion (page16, bottom paragraph, page 17, top paragraph). In fact, the mechanisms involved in the *coordinate* control of the two key determinants of skeletal muscle fitness: mitochondrial function and contractile fiber type remain largely unclear in the whole field. Our findings that muscle FNIP1 orchestrates mitochondrial function and type I muscle fiber program through both AMPK-dependent and independent signaling is an important novel aspect in this regard.

The study by Reyes et al. (Proc Natl Acad Sci U S A., 2015, 112, 424-429) reported that whole body *Fnip1* knockout mice exhibit increased proportion of type I fibers and resistant to muscle fatigue. While this suggests a role for FNIP1 in type I muscle fiber specification, the intrinsic roles of muscle FNIP1 in coordinating mitochondrial program and muscle fiber type, the two interrelated but distinct processes in skeletal muscles, is not understood. Moreover, the Reyes et al study was performed by whole body deletion of *Fnip1*, the global *Fnip1* KO have very complicated phenotype. For instance, in addition to muscle changes, whole-body FNIP1 deficiency has profound effects on immune cell development, cardiomyopathy, and kidney development and function (Park et al., Immunity, 2012, 36, 769-781; Park et al., Proc Natl Acad Sci U S A., 2014, 111, 7066-7071; Reyes et al., Proc Natl Acad Sci U S A., 2015, 112, 424-429; Siggs et al., Proc Natl Acad Sci U S A., 2016, 113, E3706-3715; Centini et al., PLoS One, 2018, 13, e0197973). It is worth noting that according to published debate (Handschin and Spiegelman, Cell Metab. 2011, 13, 351; Zechner et al., Cell Metab., 2011, 13, 352), the solely use of a global *Fnip1* KO model could result in confounding secondary effects driven by FNIP1 deficiency in nonmuscle tissues. Indeed, reports on the effects of FNIP1 on both AMPK and mTOR have been inconsistent and, thus, are less conclusive (Reyes et al., Proc Natl Acad Sci U S A., 2015, 112, 424-429; Hasumi et al., Proc Natl Acad Sci U S A., 2015, 112, E1624-1631; Petit et al., J Cell Biol., 2013, 202, 1107-1122; Ramirez et al., J Immunol., 2019, 203, 2899-2908). Therefore, the intrinsic physiological role of FNIP1 in skeletal muscle remains unclear. In this study, we used both gain-of-function and loss-of-function strategies in mice to generate a series of genetically modified mouse lines, including *Fnip1*-knockout (*Fnip1*^{KO}) mice and *Fnip1*^{TgKO} mice expressing *Fnip1* only in skeletal muscle but not in other tissues. Our mouse and muscle cell genetic systems afforded us the unique opportunity to define the intrinsic role of FNIP1 in skeletal muscle. We provided clear genetic data that led to the following surprising conclusions, which are different from the previous reports: 1) Basal levels of FNIP1 are sufficient to function as a "brake" to simultaneously inhibit mitochondrial function and type I fiber program in skeletal muscle, while muscle

function is normal and fiber types are unchanged in muscle-specific *Fnip1* transgenic mice; 2) FNIP1 in fact cell-autonomously regulates AMPK but not mTORC1 signaling in skeletal muscle cells, thus shed light on the inconsistencies in previous data; 3) FNIP1 regulates muscle mitochondrial oxidative program through AMPK, these findings are recapitulated in skeletal muscle cell culture and, thus, are cell autonomous; 4) FNIP1 regulates type I fiber program independent of AMPK/PGC-1α, mitochondrial function and muscle fiber type are indeed controlled by distinct signaling pathways. Together, we have genetically established a pivotal intrinsic role for FNIP1 in orchestrating mitochondrial function and type I muscle fiber, as well as its epistatic relationship with AMPK and PGC-1α in driving muscle fiber type determination in skeletal muscle.

Regarding the role of AMPK/PGC-1α signaling in the FNIP1-dependent fiber type determination described in this manuscript: The Hasumi et al. paper quoted in this query reported mainly on effects of FLCN on muscle color and mitochondrial biogenesis rather than muscle fiber type. In fact, a surprising finding of our study was dissociation between mitochondrial function and type I fiber program in *Fnip1*^{KO}, *Ampka1/a2*$^{f/f/Myf5\text{-}Cre}$ (TKO) and *Fnip1*^{KO}, PGC-1α$^{f/f/MCK\text{-}Cre}$ (DKO) mice muscles, and we concluded that FNIP1-dependent type I muscle fiber program does not require the AMPK/PGC-1α signaling based on the formal muscle fiber typing phenotype of our multiple genetic mouse lines: First, MHC fiber typing data from two independent *Ampka1/a2* KO lines revealed that FNIP1 deficiency induces increase proportion of type I fibers in all soleus, GC and EDL muscle in the absence of *Ampka1/a2* (original Fig 6 A-C). Second, gene expression studies (original Fig 6 D) demonstrated that the expression of the major type I myosin isoform MHC1 (Myh7 gene) and slow-twitch troponin genes was induced by FNIP1 deficiency but not affected by the complete loss of muscle *Ampka1/a2* in GC muscle. To further corroborate this point, data from additional experiments showed that the expression of slow-twitch type I muscle fiber genes was activated by FNIP1 deficiency but not affected by disruption of *Ampka1/a2* in soleus muscle (revised S8 Fig), indicating that AMPK-independent regulation of type I fiber program by FNIP1 is relevant to multiple muscle types across a range of fiber type proportions and oxidative capacity. Third, despite the complete loss of muscle PGC-1α, FNIP1 ablation can achieve type I fiber-type transformation in both soleus and GC muscle of DKO mice (original Fig 7H). It is worth noting that this is quite clear in soleus, a muscle which is enriched in PGC-1α and contains a high proportion of type I fibers. Lastly, we and others have identified independent factors, such as MEF2s and ERRs/miRNA axis, that are required for fundamental muscle fiber type determination (Potthoff et al., J Clin Invest., 2007, 117, 2459-2467; van Rooij et al., Dev Cell, 2009, 17, 662-673; Gan et al., J Clin Invest., 2013, 123, 2564-2575; Liu et al., EMBO Mol Med., 2016, 8, 1212-1228), consistent with our conclusions. This information has been added as new S8 Fig and addressed in the revised Discussion (page 18, line 389-408).

Thus, we think that our results have uncovered a previously unrecognized intrinsic role of FNIP1 that coordinately regulates mitochondrial function and muscle fiber type that govern muscle fitness. Specifically, FNIP1 regulates muscle mitochondrial program in an AMPK-dependent fashion, while it acts independently of AMPK/PGC-1α to induce type I myofiber formation. Indeed, this coordinate response is an important aspect of the message in this work, given that it could be an appealing target that coordinates multiple pathways towards reversing muscle diseases. We have clarified and emphasized these important points in the revised Discussion (page16, page 17, top paragraph, page 20, paragraph 2).

<u>Itemized Responses to Reviewer #3</u>

"Reviewer #3: The authors investigated the role of folliculin interacting protein 1 (Fnip1) in skeletal muscle phenotypes using transgenic and muscle-specific knockout mice. They report that Fnip1-KO induced a glycolytic-to-oxidative shift of the muscular metabolic profile via AMPK activation. Various experimental approaches were used, and the results are clear at a glance. The design of the experiments, however, has serious concerns."

We wish to thank the Reviewer for the critical and constructive review. Our itemized responses are as follows:

1. *"a. The authors analyzed various indexes using several kinds of hindlimb skeletal muscles. The phenotypes of mammalian skeletal muscles vary: fast-twitch and glycolytic muscle fibers dominate in the lateral gastrocnemius, EDL, and vastus lateralis muscles; slow-twitch oxidative fibers dominate in the solus and medial gastrocnemius (GAS) muscles; and fast and glycolytic fibers dominate in the TA muscle, although slow and oxidative fibers dominate in the deep portion. The role of Fnip1 may therefore differ from one mammalian muscle type to another.*
   b. The volume of skeletal muscle available for the experiments may have fallen short of the authors' requirements. The authors wanted to analyze various indexes using many kinds of skeletal muscle. The responses to Fnip1-KO in fast-type muscle, however, may differ from those in slow-type muscle, given that the nervous and endocrine systems may also mediate the muscle fiber type. The authors should analyze various indexes using the same kind of skeletal muscle.
   c. Many of the analyses were carried out using the GAS muscle. This GAS is big, hence the contractile and metabolic properties of the muscle depend on the region examined. The sizes of the sampled muscles and the actual portions of the samples are left unstated in the manuscript. The paper should clearly describe the region of the GAS muscle samples in three dimensions."

We think the Reviewer has raised important points. We agree with the Reviewer and have addressed these points experimentally and revised the manuscript to make those points clearer:

a) The Reviewer is correct. The contractile and energy metabolic properties of the muscle differ between the different muscle types. We also agree that effect of *Fnip1*^{KO} may not the same between the different muscle types. In this study, we wanted to determine whether the concerted regulation of mitochondrial and type I muscle fiber programs by FNIP1 is a broader effect and not just restricted to specific muscle type, we thus analyzed type I muscle fibers and mitochondrial oxidative metabolism readouts in multiple muscle types of *Fnip1*^{KO} mice. Interestingly, whereas we found that FNIP1 simultaneously regulates mitochondrial oxidative program and type I muscle fibers in multiple muscle types, we did observed difference in the effect of *Fnip1*^{KO} on MHC2a and MHC2x gene expression in soleus and gastrocnemius (GC) muscle (original Fig 6D and new S8 Fig), suggesting a distinct role of FNIP1 in regulating MHC2a and MHC2x expression in different muscle types. It is possible this could reflect the intrinsic differences between muscles at the baseline that affect the adaptive range of MHC transformations (Schiaffino and Reggiani, Physiol Rev., 2011, 91, 1447-1531). Please also see our response in bellow#4, we have included a new Figure (new S8 Fig)

for the expression of MHC and slow/fast-twitch troponin genes in *Fnip1*^{KO} soleus muscle and added a discussion to the revised Discussion (page 19, line 413-419).

b) The majority of the *Fnip1*^{KO} muscle phenotypic and molecular endpoints, including RNA-seq profiling and RNA analyses, Immunoblotting studies, MHC immunostaining, SDH staining, and primary muscle cells isolation were all conducted in GC muscle as it contains both the fast-glycolytic and slow-oxidative muscle fibers, thus the GC muscle has been a little bit limited because we used the entire GC muscle for each kind of studies to keep consistent between different mice. We have conducted more experiments using *Fnip1*^{KO} GC muscles and demonstrated that the protein levels of OPA1, MFN1, Myoglobin and Cyt C were all increased in GC muscle (similar to other muscle type) of *Fnip1*^{KO} mice (new S5A and S5C Fig). In addition, given that the whole GC muscle is big and not suitable for the O2K-based permeabilized muscle OCR measurements, we have provided further experiments demonstrating that both pyruvate- and succinate-driven mitochondrial respiration rates were also induced in the plantaris part of the gastrocnemius/plantaris complex of *Fnip1*^{KO} mice (new S5B Fig). These new analyses further demonstrated that FNIP1 regulates mitochondrial oxidative program in multiple muscle types. We have added these data in new S5 Fig.

c) We thank the Reviewer. GC muscle is big and heterogeneity and we did not present our data clearly, we have stated the sizes and actual portions of the GC muscle samples in both Figure legends and the Methods (line 547, 569, 917, 942, 950, 957, 1035, 1041, 1048, 1054, 1063, 1066, 1074, 1082, 1090, 1098, 1113, 1121, 1133-1135). Specifically, we did not dissect the red part from the white part of the GC muscle, and we used the entire GC muscle for various analyses, including RNA-seq profiling, RT-PCR analyses and immunoblotting studies, to make sure we are consistent between different mice. For fiber type determination and quantification, we focused on the red zone area of the medial head in the widest part of the GC muscle. We have provided more details with regard to histologic analyses in the Methods (page 23 and page 24, line 520-543). Briefly, the gastrocnemius and plantaris of each mouse were dissected as a whole and frozen in isopentane that had been cooled in liquid nitrogen. We cut 10 μm-thick serial GC muscles cross-sections from the knee cut side in a Leica CM1850 cryostat at -20 ℃ and mounted them on positively charged glass slides. Transverse sections were collected from the widest part (mid-belly) of the GC muscle, and we examined slides under the microscope during the cutting process to make sure that we are consistent in comparing the widest part of the GC muscle from mouse to mouse. We have also added a new Figure (new S6A Fig) showing the entire cross sections of the mid-belly GC muscle (including the plantaris, medial, and lateral parts) of WT, *Fnip1*^{KO} and *Fnip1*^{TgKO} mice.

2. *"Many studies have been done on the regulatory mechanisms of myosin heavy chain (MyHC) phenotypes. Previous reports have proposed that MyHC is mediated not only by PGC1-a and MAPK, but also several molecules such as calcineurin, the NFAT family, miR-208b, miR-499, and HSF1. The authors should discuss the relationship between Fnip1-AMPK signals and these candidates."*

We thank the Reviewer's insightful point. In addition to the AMPK and PGC-1α signaling pathways, previous studies have identified multiple transcriptional signaling, such as calcineurin/NFAT,

HDAC/MEF2, nuclear receptor/miRNA axis, and HSF1 in the regulation of type I muscle fiber specification (Bassel-Duby and Olson, Annu Rev Biochem., 2006, 75, 19-37; Potthoff et al., J Clin Invest., 2007, 117, 2459-2467; van Rooij et al., Dev Cell, 2009, 17, 662-673; Gan et al., J Clin Invest., 2013, 123, 2564-2575; Liu et al., EMBO Mol Med., 2016, 8, 1212-1228; Yokoyama et al., Acta Physiol., 2016, 217, 325-337). We are very interested in the relationship between FNIP1 and those factors known to regulate type I muscle fibers. We have conducted further comparative analysis of our RNA-seq data and found that *Esrrb*, *Esrrg*, *Myh7b/miR-499 and Myh7/miR-208b* mRNA levels were increased in *Fnip1*^{KO} muscle (revised S3E and S3F Fig), whereas the expression of the genes encoding calcineurin, NFAT, MEF2s and HSF1 was not increased in *Fnip1*^{KO} muscle compared with that in WT controls (revised S3E Fig). While we cannot exclude the possibility that FNIP1 signaling regulates the activity of calcineurin/NFAT, HDAC/MEF2, and HSF1 signaling, these new results suggest that activation of ERRs/miRNA axis may acts in parallel with AMPK/PGC-1α signaling to induce type I myofiber in *Fnip1*^{KO} muscle. Interestingly, we have previously reported that *Fnip1* is a direct target of miR-499, acting downstream of the ERRs/miR-499 axis (Gan et al., J Clin Invest., 2013, 123, 2564-2575; Liu et al., EMBO Mol Med., 2016, 8, 1212-1228), thereby suggesting a feedback loop whereby the FNIP1 pathway described here regulates muscle fiber-type determination. This information has been added as revised S3E and S3F Fig (page 8, line 152-156) and addressed in the revised Discussion (page 18, line 398-408).

3. *"Figure 1F: The images of Fnip1-KO are strange. Very few myofibrils appear in the upper image. This image may be derived from around the subsarcolemmal region, not from the intermyofibrils. And the mitochondria in both images appear to be swelling, with wide interstitial spaces. These are abnormal ultrastructures that may be attributed to technical errors during the fixation steps. The authors should replace the images with better-quality alternatives."*

We thank the reviewer. We confirmed the TEM images in original Fig 1F shows the intermyofibrillar mitochondria in *Fnip1*^{KO} muscle. We have improved our TEM studies and replaced the original Fig 1F with low-power and high-power electron micrographs to clarify this point (revised Fig 1F). Notably, the WT and *Fnip1*^{TgKO} mice have an ordered array of small intermyofibrillar mitochondria with dense mitochondrial matrix along the Z-lines of the sarcomeres, whereas the *Fnip1*^{KO} mice have a larger shape and less dense mitochondria, which displaced the myofibrils (revised Fig 1F). These results could suggest that FNIP1 not only regulates mitochondria quantity, but also regulate mitochondrial quality. Indeed, during the review of this manuscript, a study was published reporting that FNIP1 regulates mitochondrial redox homeostasis (Manford et al., Cell, 2020, 183, 46-61.e21). It is also possible that the altered mitochondrial morphology is due to the changes in mitochondrial location (e.g. the proximity to the myofibrils triads where calcium is released from SR). This information has been added as revised Fig 1F and 1G (page 9, line 179-183) and addressed in the revised Discussion (page 20, line 434-439).

4. *"Figures 2E and 2F:*

a. As mentioned above, the contractile and metabolic properties depend on the regions of GAS examined. Both the size of the sampled muscles and the actual portions of the samples are left unstated. The paper should clearly describe the region of the GAS muscle samples in three

dimensions.

b. Judging from the immunostained images, the percent proportion of type I fibers in the soleus was underestimated in the graph. The proportions of type I soleus fibers in the wild-type, KO, and TgKO mice appear to be ~80%, ~100% and ~90%, respectively. And, how did the authors calculate the proportions of type I fibers and SDH-positive fibers in the GAS? As mentioned above, the contractile and metabolic properties depend on the regions of the GAS examined.

c. These figures also report muscle fiber atrophy in the KO mice, compared to the WT and TgKO. Why was this? The authors should validate their data by reporting both the body weights and the absolute and relative muscle weights.

d. Many unstained fibers appear in the images. Were these type 2a? If so, the authors should explain the effects of Fnip1-KO on type 2a fibers. Note also that the effects of Fnip1-KO may vary among muscle types."

We thank the Reviewer's insightful points, our responses to the each of the points are as follows:

a) We agree and this was addressed in the point#1c above. In brief, we have stated the sizes and actual portions of the GC muscle samples in Figure legends and Methods. We have also added a new Figure (new S6A Fig) showing the entire cross sections of the mid-belly GC muscle (including the plantaris, medial, and lateral parts) of WT, *Fnip1*^{KO} and *Fnip1*^{TgKO} mice.

b) As for type 1 fiber quantitation in soleus muscle, we have replaced the original Fig 2E with new representative images and re-quantified the percent proportion of type I fibers from additional narrow age range mouse soleus samples (revised Fig 2F and 2G). We confirmed a clear significant increase in type I fiber proportion in *Fnip1*^{KO} soleus as compared to WT or *Fnip1*^{TgKO} soleus muscle (WT, 37.5 ± 3.4 % vs. *Fnip1*^{KO}, 80.7 ± 7.0 %, p < 0.001; *Fnip1*^{TgKO}, 43.1 ± 4.6 % vs. *Fnip1*^{KO}, 80.7 ± 7.0 %, p < 0.001; n = 6-7 mice per group), and we have also enlarged the soleus image in original Fig 2E to clarify this point (see Figure 1 on the left). The quantification of type 1 fiber and SDH staining in GC muscle has been a challenge, in part, due to heterogeneity. We focused on the same red zone area of the medial head of GC muscle to keep consistent between different mice. We have quantified numerous sections and present this as number of type I fibers per medial head of GC (WT, 77 ± 24 per section vs. *Fnip1*^{KO}, 337 ± 73 per section, p < 0.001; n = 7-8 mice per group) and percentage of positive staining myofibers for SDH per medial head of GC (WT, 33.5 ± 7.8 % vs. *Fnip1*^{KO}, 76.9 ± 7.8 %, p

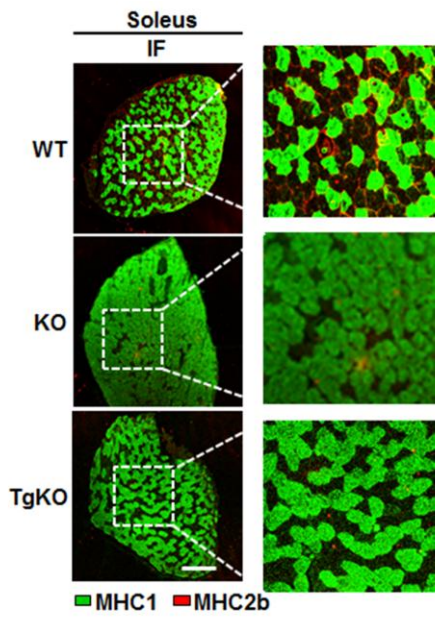

Figure 1. Cross-section of soleus muscle from WT, *Fnip1*^{KO} and *Fnip1*^{TgKO} mice stained for MHC immunofluorescence. MHC1 (green), and MHC2b (red). The panels on the right show magnified areas of the panels on the left. Scale bar: 300 µm.

< 0.001; n = 8 mice per group). We have provided more details with regard to histologic analyses in the Methods (page 23 and page 24, line 520-543).

c) We are very interested in muscle atrophy, please also see our response in bellow #7. By gross examination, hindlimb muscle of adult *Fnip1*^{KO} mice was smaller than that of WT or *Fnip1*^{TgKO} mice. We have added new supplemental table demonstrating that the body weight, weight of GC muscles and GC muscle weight normalized to body weight were decreased in *Fnip1*^{KO} mice compared to WT or *Fnip1*^{TgKO} mice at 12 weeks of age (new S1 Table). These results suggested a potential role of muscle FNIP1 in controlling body weight and muscle mass. It is possible that the difference in muscle size could reflect the fiber type switching. It is also possible that FNIP1 directly regulates the dynamic balance between protein synthesis and degradation that determine muscle mass. We wish to establish the necessary assay systems in our independent studies to address this interesting muscle atrophy mechanistic question. This information has been added as new S1 Table (page 7, line 142-144) and addressed in the revised Discussion (page 19, line 420-424).

d) The unstained fibers in the soleus images are likely type 2a given that soleus is type 1 and 2a fiber rich, whereas the unstained fibers in the medial head of GC muscle are likely type 2a and 2x. Unfortunately, we have not been very successful when trying to conduct type 2a and 2x immunostaing for technical reasons. However, as shown in the quantitative RT-PCR data in original Fig 6D, MHC2a (*Myh2* gene) and MHC2x (*Myh1*) mRNA were significantly increased in GC muscles in the *Fnip1*^{KO} mice compared to WT controls. Interestingly, we have conducted further experiments and found that the expression of MHC2a and MHC2x were actually reduced in soleus muscles of the *Fnip1*^{KO} mice (new S8 Fig). Whereas we found that the concerted regulation of mitochondrial and type I fiber programs by FNIP1 is relevant to multiple muscle types, these new data suggest that FNIP1 exerts a different control effect on MHC2a and MHC2x expression in different muscle types. It is possible this could reflect the intrinsic differences between muscles at the baseline that affect the adaptive range of MHC transformations (Schiaffino and Reggiani, Physiol Rev., 2011, 91, 1447-1531). This information has been added as new S8 Fig and addressed in the revised Discussion (page 19, line 413-419).

5. *"Figures 3F and 5D: Why did the authors evaluate the mitochondrial density using myoblasts instead of myotubes? All of the other analyses were performed using myotubes. Phase-contrast images should be shown."*

We also found increased mitotracker immunofluorescence signal in *Fnip1*^{KO} myotubes compared to WT or TKO myotubes. These results together with phase-contrast images of myotubes were added to the new S7 Fig.

6. *"Figures 3G and 2H: The origin of the primary cells should be shown."*

We thank the reviewer. Primary myoblasts were isolated from GC muscle of 4-week old male WT, *Fnip1*^{KO}, or TKO mice. We have provided more details with regard to primary muscle cell culture in the

Methods section (page 28 and 29, line 640-652). We have also added the origin information of the primary muscle cells to the revised Figure legend (line 968, 1012-1013, 1142).

7. "Figures 6A and 7H:

a. Judging from the images, Fnip1-KO induced muscle atrophy in the soleus and EDL in Figure 6A and in the GAS in Figure 7H. The results vary from figure to figure. The authors should explain this variation. Showing a cross-sectional area of the fiber in each condition would help to validate the data.

b. According to the figures, Fnip1-KO changed the proportion of unstained fibers. Why was this? And again, as mentioned, the contractile and metabolic properties depend on the regions of the GAS examined. The manuscript offers no description of either the sizes of the sampled muscles or the actual portions of the samples. The paper should clearly describe the region of the GAS muscle samples in three dimensions.

We thank the Reviewer's insightful points, our responses to the each of the points are as follows:

a) We agree that while the image data showing a clear and significant difference in type I fibers in *Fnip1*^{KO} muscle, whether FNIP1 also regulate muscle atrophy remain unclear. We have investigated further, and we believe that the potential variation between images could reflect the age differences of mice used for the original histology study. We used a wider age range of mice for the immunostaing at the beginning of the study. We have now conducted more muscle fiber typing studies with narrow age ranges mouse muscle samples. We have replaced the original images in Fig 2E, 6A and 7H with the image results from 8-9 week old male mice to clarify this point (revised Fig 2F, 6A and 7H). We have also quantified the fiber size distribution from numerous cross-sections of the soleus, EDL and the red zone area of the medial head of GC muscle as suggested. Whereas fiber-size distribution was altered in EDL muscle from *Fnip1*^{KO} mice, with a clear shift toward a reduced cross-sectional area relative to that of WT controls, no clear changes in fiber size distribution was observed in soleus and the red zone area of the medial head of GC muscle from *Fnip1*^{KO} mice (see Figure 2 below). Given that fiber size is in general highly related to the fiber type switching, the different fiber size change results may depend on the muscle region examined. Thus, we think that the data is not conclusive, so we hesitate to include these results in the revised manuscript. Whether FNIP1 regulate muscle atrophy is surely a very important and intriguing question that warrants serious further characterizations, as we have found that the muscle weight was decreased in *Fnip1*^{KO} mice compared to WT controls (new S1 Table). Please also see our response to the point#4 above. We primarily focused on the coordination of type I muscle fiber and mitochondrial programs by FNIP1 in this study, it is possible that FNIP1 directly regulates the dynamic balance between protein synthesis and degradation and is independent of the change in fiber type. We wish to establish the necessary assay systems in our independent studies to address this interesting muscle atrophy mechanistic question. This information has been added as new S1 Table (page 7, line 142-144) and addressed in the revised Discussion (page 19, line 420-424).

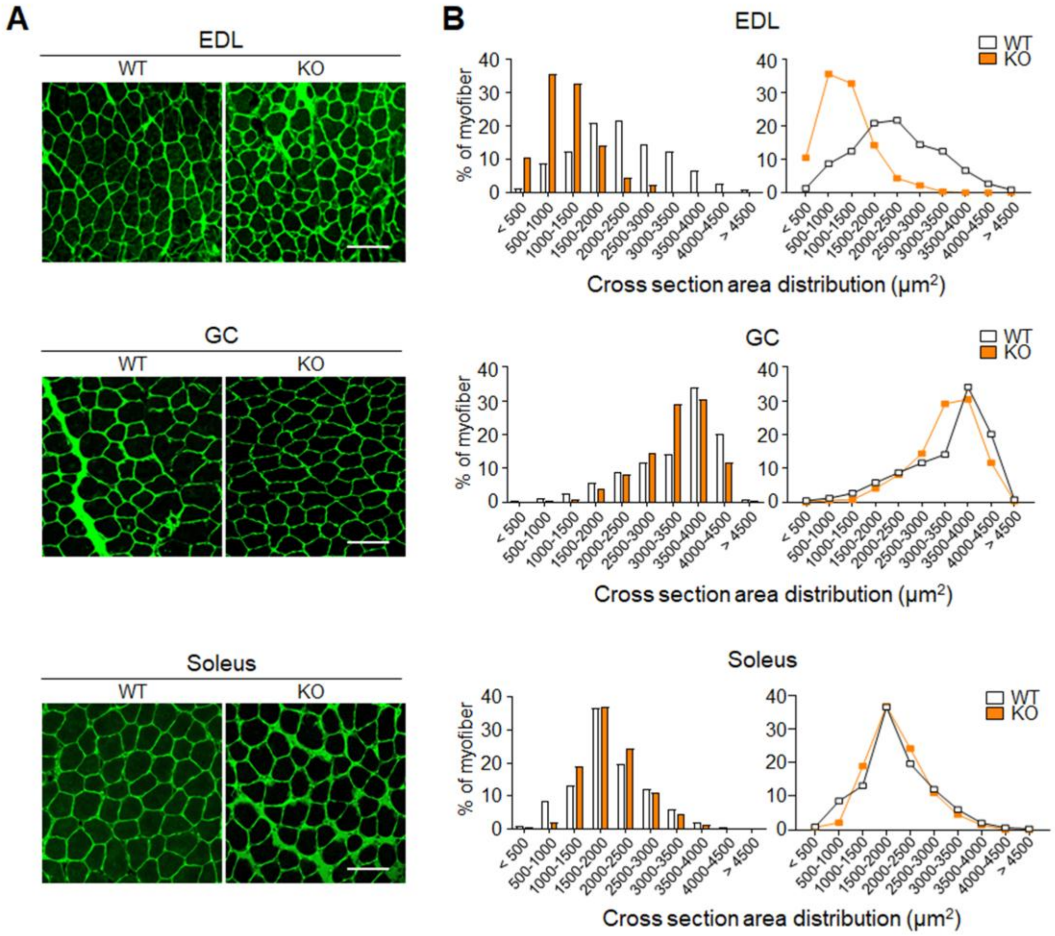

Figure 2. Muscle fiber size distribution of *Fnip1*^{KO} mice. (**A**) Representative wheat germ agglutinin (WGA) staining of EDL, red zone area of the medial head of GC and soleus muscle from 8-9-week-old male WT and *Fnip1*^{KO} mice. Scale bar: 100 µm. (**B**) Cross-sectional areas of myofibers were measured by ImageJ, 300-700 myofibers from each group were measured, n = 5-8 mice per group.

b) The unstained fibers in *Fnip1*^{KO} muscle and the sizes and actual portions of the GC muscles were addressed in the point#1 and #4 above. In brief, the unstained fibers in the soleus images are likely type 2a, whereas the unstained fibers in medial head of GC images are type 2a and 2x. We have added a discussion to the revised Discussion (page 19, line 413-419). In addition, we have stated the sizes and actual portions of the GC muscle samples in Figure legends and Methods. We have also added a new Figure (new S6A Fig) showing the entire cross sections of the mid-belly GC muscle.

8. *"Figure 7B: The authors failed to detect PGC-1a by Western blotting. And, what does "n.s." stand for here? The authors should provide a detailed description of the procedure for their primary cell culture experiments. How long were the cells incubated with the differentiation medium? Note that the differentiation level may affect the metabolic profiles"*

We appreciate the Reviewer's point. We think it is worth noting that PGC-1α protein levels are hardly detectable in WT GC muscle. *n.s.* in the original Fig 7B stands for "none-specific band". We have improved the PGC-1α Western blotting and replaced the original Fig 7B with the new immunoblot analysis of PGC-1α using whole cell extracts from entire GC muscle and heart from indicated mice (revised Fig 7B). The data now indicate a clear increase in the expression of PGC-1α protein in *Fnip1*^{KO} muscle, and the induced expression of PGC-1α protein was completely abolished in the DKO muscle (see Figure 3 below).

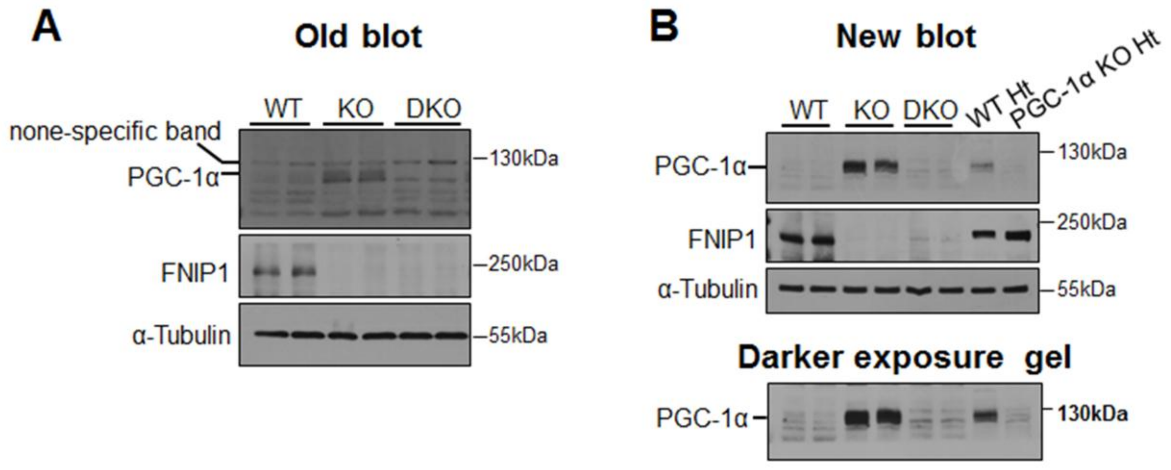

Figure 3. PGC-1α protein levels is increased in *Fnip1*^{KO} muscle. (**A**, **B**) Immunoblot analysis of PGC-1α using whole cell extracts from WT and PGC-1α KO heart (positive and negative control) and gastrocnemius muscle fromWT, *Fnip1*^{KO}, *Fnip1*^{KO}/PGC-1α$^{f/f/MCK\text{-}Cre}$ (DKO) mice.

We have provided more details with regard to primary muscle cell culture in the Methods section (page 28 and 29, line 640-652). In briefly, primary myoblasts were isolated from GC muscle of 4-week old male mice as previously described (Rando and Blau, J Cell Biol., 1994, 125, 1275-1287; Gan et al., J Clin Invest., 2013, 123, 2564-2575; Liu et al., EMBO Mol Med., 2016, 8, 1212-1228; Fu et al., Cell Rep., 2018, 23, 1357-1372). GC muscles from both legs were removed. Minced tissue was digested in a collagenase/dispase/$CaCl_2$ solution for 1.5 hours at 37 ℃ in a shaking bath. DMEM supplemented with 10% FBS (PPM) was added and samples were triturated gently before loading onto a Netwell filter (70 µm, BD). Cell suspension was pelleted at 1000 rpm for 5 minutes. Cells were then resuspended in PPM and plated on an uncoated plate for differential plating. Cell suspension (not-adherent) was centrifuged for 5 minutes at 1000 rpm and pellet was resuspended in Growth Medium (GM) (Ham's F-10 medium supplemented with 20% FBS and 2.5 ng/ml bFGF). Cells were plated on collagen coated flasks for expansion. Cells were fed daily with GM. For differentiation, cells were washed with PBS and re-fed with 2% Horse-serum/DMEM differentiation medium and re-fed daily. Cells were induced to differentiation for 3 days prior to various experiments, and no notable difference in myotube differentiation between *Fnip1*^{KO} myocytes and WT controls were observed (new S7 Fig). We have added the differentiation days to the revised Figure legend (lines 968, 1013 and 1143) and phase-contrast images of myotubes to the new S7 Fig.

9. *"Figures 3G and 5E: What kind of analysis was applied for the statistical analyses? This type of data should be analyzed using 2-way ANOVA."*

The original data in Fig 3G and 5E were analyzed by one-way ANOVA, and we have re-analyzed those seahorse data using two-way ANOVA and Bonferroni post-hoc test and added a sentence to the revised Figure legend (revised Fig 3G and 5E, lines 983-984, 1026-1027). Thanks.

10. *"Lines 130-131, 136-138: At this point, none of the experimental data support a glycolytic-to-oxidative shift of skeletal muscles in Fnip1-KO mice. The result merely shows a shift in the color of the muscle from whitish to red"*

Agree. We have removed those sentences, thanks.

11. *"Line 133: "FLCN" should be spelled out. "IF" should be spelled out."*

We thank the reviewer and Folliculin (FLCN) and Immunofluores (IF) have been defined in the text and Figure legend (lines 91, 956, and 1034).

12. *"a-tubulin is unsuitable for the loading and transfer control of Western blotting."*

We thank the reviewer. For the loading and transfer control of Western blotting, we have used α-tubulin for quite a few years in our lab because we find it is stably expressed and least variable across many tissues and cell lines we tested. Of note, we have also conducted β-actin and Ponceau red staining for the loading or transfer control in Western blotting (revised S5C Fig), and we see a similar result. Therefore, we are confident in our Western blotting conclusion.
