# Supplementary material for: AMPK-dependent and -independent coordination of mitochondrial function and muscle fiber type by FNIP1
Source: PLoS Genet. 2021 Mar 29;17(3):e1009488. doi: 10.1371/journal.pgen.1009488 (PMC8031738; doi:10.1371/journal.pgen.1009488)
Supplement: Attachment — Submitted filename: PGENETICS-D-20-01073R2_Itemized Response to Reviewers.pdf [file pgen.1009488.s016.pdf]

Itemized Responses to Reviewer #1

> *"Reviewer #1: The authors addressed all my concerns, and made a relevant effort to improve the manuscript. I don't have any further question at this point."*

We thank this reviewer for a comprehensive and insightful review.

Itemized Responses to Reviewer #3

We thank this reviewer for a comprehensive and insightful review. Responses to the each of the comments are as follows:

> *"The authors have revised their manuscript in response to my previous comments. Though several points of concern have been addressed in the revised manuscript, a few others still require attention."*

1. *"Figure 1F:*
   As I pointed out, the EM images are unusual. The new images added as replacements, moreover, are of poor quality.
   The replacement images of Fnip1-KO are still unusual. Very few myofibrils appear in the upper image, and those that do are atrophied. If the myofibrils are in fact as scarce and atrophied as they appear, the skeletal muscle may develop very low force, if any. The width of the I-band, moreover, is too narrow, and the mitochondria appear to be swelling, with wide interstitial spaces.
   The new images of TgKO are also unusual, showing shortened sarcomeres. These are abnormal ultra-structures that may have resulted from technical errors during the fixation steps. The authors should replace the images with better-quality alternatives."

We thank the reviewer. As suggested, we have conducted additional EM experiments to further optimize our EM fixation procedure. We think the reviewer is correct and we were able to reduce the abnormal ultrastructures and significantly improve our mitochondrial EM image quality based on our new EM protocol. We have replaced the original Figure 1F with the new mitochondrial EM image results to clarify this point (revised Fig 1F). Our new EM analysis confirmed a hyperproliferation of mitochondria with normal cristae in the *Fnip1*^{KO} mouse muscle. The mitochondria in the skeletal muscles of *Fnip1*^{KO} mice were more numerous and often significantly larger than the WT controls or *Fnip1*^{TgKO} muscles. Notably, in some regions, the marked mitochondrial proliferation appeared to have replaced the myofibrillar contractile apparatus. As addressed in our response to the original review, and in the Discussion of the revised manuscript, we believe that studies aimed at defining the role of FNIP1 signaling in myofiber atrophy would be an important focus of study for the future. This information has been added as revised Fig 1F (page 9, line 182-183) and addressed in the revised Discussion (page 19, line 420-423). We have also updated the EM analyses in the Methods (page 26, line 579-589).

2. *"Figures 3F and 5D:*
   The authors added MitoTracker-stained images of myotubes as the new Fig. S7. They did not, however, make any evaluations on the mitochondrial density of these images. As I pointed out, the mitochondrial density should be assessed using myotubes instead of myoblasts. What, specifically, does the "higher mitochondrial potential" in in the myoblasts describe? Myoblasts cannot be representative of myofibers."

We thank the reviewer. As suggested, we have added the quantification of mitotracker fluorescent signal intensity in myotubes to the revised S7D Fig. The data indicate an increase in mitochondrial mass in the *Fnip1*^{KO} myotubes as compared to WT controls or TKO myotubes (revised S7D Fig), which was

consistent with mitochondrial respiration function measurements (revised Fig 5D and 5E). We think we did not describe our data clearly, our data in original Fig 3F and 5D showed that mitotracker fluorescence signal was increased in *Fnip1*^{KO} myoblasts compared to WT or TKO myoblasts. These data suggest that FNIP1 deficiency induces an early mitochondrial adaptive reprogramming in myoblasts. We agree with the reviewer that myoblasts are not the same with myotubes, and we have removed the myoblast results in the original Fig 3F and 5D as suggested. The new results were added in the revised S7D Fig (page 13, line 283-285, and in the Methods, page 27, line 603-606).

3. *"The authors added a Ponceau-stained image as a transfer and internal control for Western blotting in only one figure, Fig. S5C. Do the other Western blotting images need Ponceau-stained image?"*

We thank the reviewer. As suggested, we have added additional Ponceau red staining results to revised S5D Fig (muscle samples from WT, *Fnip1*^{KO} and *Fnip1*^{TgKO} mice), revised S7A Fig (muscle samples from WT, KO and TKO mice), revised S7B Fig (WT, KO and TKO myotube samples), and revised S9 Fig (muscle samples from WT, KO and DKO mice). These new results further confirmed an AMPK/PGC-1α-dependent regulation of mitochondrial oxidative program by FNIP1. Notably, our Western blotting studies using Ponceau red staining or α-tubulin as loading control leads to the same conclusion. In fact, we have used α-tubulin as the loading and transfer control for Western blotting for quite a few years in our lab because we find it is stably expressed in muscle and not variable across many mouse lines we examined (Liu et al, *J Clin Invest*. 2020; 130(9):4710-4725; Fu et al, *Cell Rep*. 2018; 23(5):1357–1372; Liu et al, *EMBO Mol Med.* 2016; 8(10):1212-1228; Liang et al, *J. Biol. Chem.* 2016; 291(49):25306-25318). Based on our own experience, we have not found evidence to suggest that muscle α-tubulin is variable and not suitable for Western blotting control. Of note, equal total protein was loaded to each lane for Western blotting comparison. We have also added sentences to the Methods to clarify this point (page 28, line 629-632).

Itemized Responses to Large-Scale Datasets and Numerical Data

> *"**Have all data underlying the figures and results presented in the manuscript been provided?** Large-scale datasets should be made available via a public repository as described in the PLOS Genetics data availability policy, and numerical data that underlies graphs or summary statistics should be provided in spreadsheet form as supporting information.*
>
> *Reviewer #1: Yes*
>
> *Reviewer #3: None"*

The RNA-seq data discussed in this manuscript have been deposited in the NCBI Gene Expression Omnibus and are accessible through GEO Series accession number GSE150193 with the secure token: qtifesasnjgbvaj. This information was provided in the original manuscript in the Materials and Methods section, page 22 (revised manuscript page 25, line 564-567). In addition, all the numerical data was provided in revised S1 Data.
